# Decoupling of mechanical properties and ionic conductivity in supramolecular lithium ion conductors

**DOI:** 10.1038/s41467-019-13362-4

**Published:** 2019-11-26

**Authors:** David G. Mackanic, Xuzhou Yan, Qiuhong Zhang, Naoji Matsuhisa, Zhiao Yu, Yuanwen Jiang, Tuheen Manika, Jeffrey Lopez, Hongping Yan, Kai Liu, Xiaodong Chen, Yi Cui, Zhenan Bao

**Affiliations:** 1 Department of Chemical Engineering, Stanford University, Shriram Center, 443 Via Ortega, Room 307, Stanford, CA 94305 USA; 2 School of Chemistry and Chemical Engineering, Shanghai Jiao Tong University, Shanghai, 200240 China; 3 Department of Polymer Science and Engineering, School of Chemistry and Chemical Engineering, Nanjing University, Nanjing, 210093 P. R. China; 4 Innovative Center for Flexible Devices (iFLEX), School of Materials Science and Engineering, Nanyang Technological University, 50 Nanyang Avenue 639798, Singapore, Singapore; 5 Department of Materials Science and Engineering, Stanford University, 476 Lomita Mall, Stanford, CA 94305 USA; 6 Stanford Institute for Materials and Energy Sciences, SLAC National Accelerator Laboratory, 2575 Sand Hill Road, Menlo Park, CA 94025 USA

**Keywords:** Mechanical properties, Polymer characterization, Supramolecular polymers, Batteries, Batteries

## Abstract

The emergence of wearable electronics puts batteries closer to the human skin, exacerbating the need for battery materials that are robust, highly ionically conductive, and stretchable. Herein, we introduce a supramolecular design as an effective strategy to overcome the canonical tradeoff between mechanical robustness and ionic conductivity in polymer electrolytes. The supramolecular lithium ion conductor utilizes orthogonally functional H-bonding domains and ion-conducting domains to create a polymer electrolyte with unprecedented toughness (29.3 MJ m^−3^) and high ionic conductivity (1.2 × 10^−4^ S cm^−1^ at 25 °C). Implementation of the supramolecular ion conductor as a binder material allows for the creation of stretchable lithium-ion battery electrodes with strain capability of over 900% via a conventional slurry process. The supramolecular nature of these battery components enables intimate bonding at the electrode-electrolyte interface. Combination of these stretchable components leads to a stretchable battery with a capacity of 1.1 mAh cm^−2^ that functions even when stretched to 70% strain. The method reported here of decoupling ionic conductivity from mechanical properties opens a promising route to create high-toughness ion transport materials for energy storage applications.

## Abstract (web summary)

Typically, ion conducting polymers exhibit a trade-off between mechanical robustness and ionic conducting performance. Here, the authors utilize supramolecular chemistry obtaining extremely tough electrolytes with high ionic conductivity and enabling stretchable lithium-ion batteries.

## Introduction

Lithium ion batteries (LIBs) are relied upon to provide energy storage for electric vehicles, grid-level storage, personal electronics, and increasingly, wearable soft electronics that interface with the human body^1–3^. Unfortunately, ion transport in modern LIBs relies on flammable liquid electrolytes, which are culpable for the majority of recent catastrophic battery fires^4^. Developing solid electrolyte materials is critical to meet the performance demands of modern LIBs while providing requisite increases in mechanical stability, thermal stability, and safety^5^. As applications for batteries for wearable and conformable electronics continue to emerge^6–8^, the growing intimacy between batteries and the human body exacerbates the need for improved robustness. The development of solid electrolyte materials for batteries requires creation of materials that are both highly ionically conductive and mechanically robust.

While ceramic ion conductors offer high ionic conductivity, they are brittle and difficult to implement, and so polymer electrolytes have been pursued as a means to increase safety while maintaining low cost and easy processability^9^. It is well known that ion transport in polymer electrolytes is governed by the Vogel-Tamman-Fulcher (VTF) relationship. The VTF equation dictates that a lower T_g_ in a polymer electrolyte leads to higher ionic conductivity^10^. Because of this, a preponderance of polymer electrolyte research in the past 40 years has focused on reducing the T_g_ of polymer electrolytes in order to improve ionic conductivity^11–13^. Unfortunately, lowering the T_g_ of a polymer is deleterious to its mechanical strength^14^, and so a polymer electrolyte with a low T_g_ could lead to hazards such as short circuiting via external puncture or from dendrite formation^15^. As such, developing polymer electrolytes with good mechanical strength and good ionic conductivity remains a challenge.

To avoid the canonical trade-off between ionic conductivity and mechanical properties in polymer electrolytes, several polymer engineering strategies have been employed. The most eminent strategy is based on a polystyrene(PS)-polyethylene oxide(PEO) block copolymer, in which the PS block provides mechanical strength and the PEO block provides ionic conductivity^16,17^. Other strategies include nanoscale-phase separation^18^, crosslinking with hairy nanoparticles^19^, addition of ceramic fillers^20^, and others^21,22^. However, to date, all of these strategies result in rigid electrolytes, and thus none are suitable for applications in which conformable and stretchable batteries are needed. Current stretchable batteries rely on mechanically weak gel electrolytes^23,24^ or on strain engineered structures incorporating liquid electrolyte^25,26^, which present unacceptable safety hazards for use in wearable technology.

In this work, we demonstrate an effective method of decoupling ionic conductivity from mechanical properties in polymer electrolytes. We have designed a supramolecular lithium ion conductor (SLIC) in which ionic conductivity is provided by a low-T_g_ polyether backbone and mechanical properties are provided by the dynamically bonded 2-ureido-4-pyrimidone (UPy) backbone unit. Because the ion transport in the soft segment is governed by the T_g_ of the polyether and the mechanical properties arise orthogonally from the UPy group^27^, we obtain a polymer electrolyte with an unprecedented toughness of 29.3 ± 1.4 MJ m^−3^ and a high ionic conductivity of 1.2 ± 0.21 × 10 ^−4^ S cm^−1^ at room temperature. This is the first work in which supramolecular design is used to provide decoupling of T_g_ from toughness in a polymer electrolyte.

To further show the utility of the SLIC material in battery applications, we demonstrate that the mechanical strength and ionic conductivity of SLIC make it an excellent binder material for stretchable LIB electrodes. We show that intrinsically stretchable electrodes with strain capability of up to 100% can be obtained through a conventional slurry-casting process when as little as 20 wt. % SLIC is used as a binder. Furthermore, we show that the dynamic nature of the supramolecular binder and electrolyte allows for the formation of a strong and continuous electrode-electrolyte interface. We briefly demonstrate the ability to combine the SLIC electrolyte and SLIC electrodes to create a LIB that is intrinsically stretchable on the molecular level^28^. Our approach to fabricate stretchable LIBs based on stretchable supramolecular materials offers potential advantages in processability and energy density compared to previously reported strain-engineering approaches. The strategy reported here of using supramolecular dynamic bonding to create stretchable ion conductors opens a promising avenue for fabricating tough materials for intrinsically stretchable energy storage devices.

## Results

### Characterization of supramolecular SLIC polymers

**Fig. 1:**
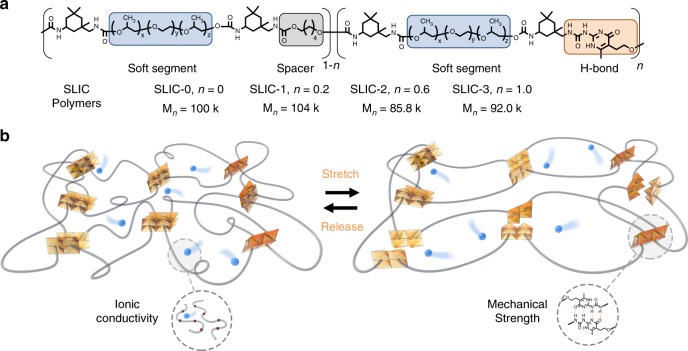
Schematic of the SLIC macromolecules presented in this work. **a** Chemical structure of SLIC and the composition and molecular weight of SLIC-0 to SLIC-3. x = 9, y = 23, z = 9. **b** Diagram showing the general operating principle of a SLIC-based polymer electrolyte upon stretching. Blue circles represent lithium ions, black wires are PPG-PEG-PPG chains, and orange squares are hydrogen-bonding UPy moieties.

Figure 1a shows a schematic of the synthesized SLIC macromolecules. As shown in Supplementary Fig. 1, SLIC molecules were synthesized through condensation of hydroxy terminated macromonomers, UPy precursors, and diisocyanate linkers. The SLIC macromolecule contains a soft segment based on the ion-conducting polymer poly(propylene glycol)-pol(ethylene glycol)-poly(propylene glycol) (PPG-PEG-PPG). The PPG-PEG-PPG block was chosen for ease of synthesis and to eliminate conductivity-reducing crystallization of the PEG soft segment in the final polymer^29^. The molecular weight of the soft segment is *ca*. 2000 Da. To impart mechanical strength to the polymer, the strong quadruple hydrogen-bonding motif 2-ureido-4-pyrimidone (UPy) is included in the backbone^30^. The strong association constant between UPy moieties makes the bonds almost as strong as covalent bonds while retaining dynamic properties due to the reversible nature of the hydrogen bonds^31,32^. An aliphatic extender is also included to enable modification of the amount of UPy while keeping the soft segment concentration constant. To systematically investigate the effect of the hydrogen-bonding UPy moiety on the mechanical properties and ion-transport properties of the macromolecules, a series of polymers denoted SLIC-0, SLIC-1, SLIC-2, and SLIC-3 were synthesized. SLIC-0 contains 0% hydrogen-bonding units in the backbone, whereas SLIC-3 contains 100% UPy and no aliphatic extenders. The molecular weights of the synthesized SLICs are around 100 kDa as determined by GPC. ^1^H NMR confirms successful synthesis of the SLIC molecules (Supplementary Figs. 2–5). Figure 1b shows a schematic of the operating principle of the SLIC macromolecules. In SLIC, lithium ions are transported through the PPG-PEG-PPG soft segment, which makes up the majority (~77 mol. %) of the polymer. The UPy groups in the polymer backbone interact with each other, creating high mechanical strength. When stretched, the polymer can mechanically dissipate stress through reversible breakage of the H-bonds while maintaining the ion-transport pathways.

**Fig. 2:**
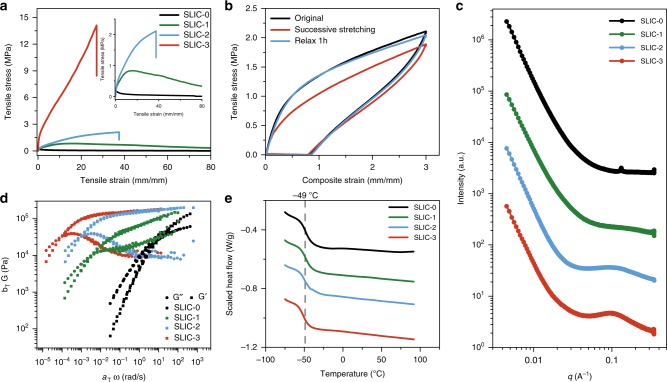
Characterization of SLIC macromolecules. **a** Stress-strain curves of SLIC-0 to SLIC-3 at an extension rate of 100 mm min^−1^. Inset: zoom in of low-stress region of the stress-strain curve. **b** Strain cycling of SLIC-3 at a rate of 30 mm min^−1^. SLIC-3 is stretched to 300%, and then stretched again immediately. After relaxing 1 h, the third stretch is performed. **c** SAXS of SLICs. **d** Time-temperature superposition rheology of SLIC-0 to SLIC-3. **e** DSC Traces of SLICs. The constant T_g_ at around −49 °C is indicated.

The mechanical properties of the as-synthesized SLIC molecules are of key importance when assessing the feasibility of the polymer for use as a robust stretchable electrolyte. Figure 2a shows stress-strain curves of SLIC-0 through SLIC-3. For SLIC-0, the tensile stress in the sample is extremely low, and the polymer yields at low strain. As the amount of UPy in the backbone increases, the tensile stress required to stretch the elastomers increases systematically. For SLIC-3, an impressive extensibility of ~2700 ± 63 % and an ultimate stress of 14 ± 0.2 MPa are obtained. The elastic behavior of SLIC-3 is shown in Fig. 2b. While the dynamic nature of the hydrogen-bonding crosslinks imparts viscoelastic behavior to the polymers, SLIC-3 shows excellent stress recovery at low strains upon successive cycling. After resting for 1 h, the polymer completely recovers its original mechanical properties. The cyclic stress-strain curves for SLIC-0,1,2 are shown in Supplementary Fig. 6. While these polymers are also viscoelastic, they demonstrate much lower stress recovery than SLIC-3. As expected, the ability to recover from strain increases as the amount of hydrogen bonding in the network increases. To investigate the structure of the SLIC polymers, small-angle X-ray scattering (SAXS) measurements in transmission geometry were performed (Fig. 2c). As the UPy content of the polymer increases from SLIC-0 to SLIC-3, a broad peak corresponding to a d-spacing of ~6 nm becomes more prominent. This broad scattering peak indicates the presence of phase-separated hydrogen bonding aggregates that are homogeneously distributed^33^, which leads to excellent mechanical properties of the polymer. The broadness of this peak indicates that the population of the UPy domains is low, as expected based on the relatively low concentration of UPy groups (<22 mol. %) in SLIC. Additionally, FTIR measurements confirm the presence of hydrogen bonding within the system. FTIR spectra in Supplemental Fig. 7 show an increase in intensity for peaks representing C=O H-bonding in urea (1660 cm^−1^) and C=O H-bonding in urethane (1695 cm^−1^) from SLIC-0 to SLIC-3^32^.

Figure 2d shows the rheological properties of SLIC-0 to SLIC-3. Time-temperature superposition rheometry is used to obtain data for the shear modulus of the SLIC molecules from 10^−5^ to 10^3^ rad s^−1^. From the rheology, it can be observed that the modulus for the rubbery plateau is similar for all of the SLICs. The crossover point between the loss and storage modulus is the location at which the polymer undergoes a transition from being “liquid-like” to being “solid-like.” Fig. 2d shows that as the amount of UPy in the polymer backbone increases from SLIC-0 to SLIC-3, the polymer relaxation time becomes slower, which is consistent with the increased crosslinking density that is expected from the UPy H-bonding. This also means that at short time scales, SLIC-0 will relax and ‘flow’ more than SLIC-3. Despite this viscoelastic behavior, the creep measurements in Supplementary Fig. 8 show that SLIC-3 has minimal creep at moderate strains.

Compared to other supramolecular dynamic polymers reported in the literature, SLIC has very impressive properties. While sacrificial bonding has been used in polymer electrolytes in the past, a truly supramolecular LIB polymer electrolyte has not been reported. Previous works with dynamically bonded electrolytes relied on covalent bonds to provide mechanical strength at the expense of ionic conductivity^34–36^. Supplementary Table 1 compares the SLIC polymers to various other commercial and recently reported elastic/viscoelastic polymers^37–39^. Supplementary Fig. 9 shows that when comparing the toughness and extensibility of SLIC to other polymers, SLIC is among the toughest-reported polymers and maintains impressive extensibility. These unique mechanical properties make SLIC an attractive choice for applications requiring high stretchability and toughness.

Figure 2e shows the DSC traces for SLIC-0 to SLIC-3. Importantly, the SLICs show a constant T_g_ at around −49 ^o^C. The T_g_ of supramolecular polymers originates from the local dynamics of the soft segment, and so the observed T_g_ arises from the relaxation of the soft PPG-PEG-PPG segment and is independent of the UPy content in the backbone^30,40^. This result agrees with previous observations in supramolecular polymers^27,37^. The fact that a constant T_g_ is observed despite the vastly different mechanical properties of the SLICs shows that the polymer segmental relaxation is decoupled from the toughness in this system. Overall, the supramolecular design of the SLIC material provides excellent control over the mechanical properties of the polymer system while maintaining a constant T_g_.

### SLIC as a polymer electrolyte

**Fig. 3:**
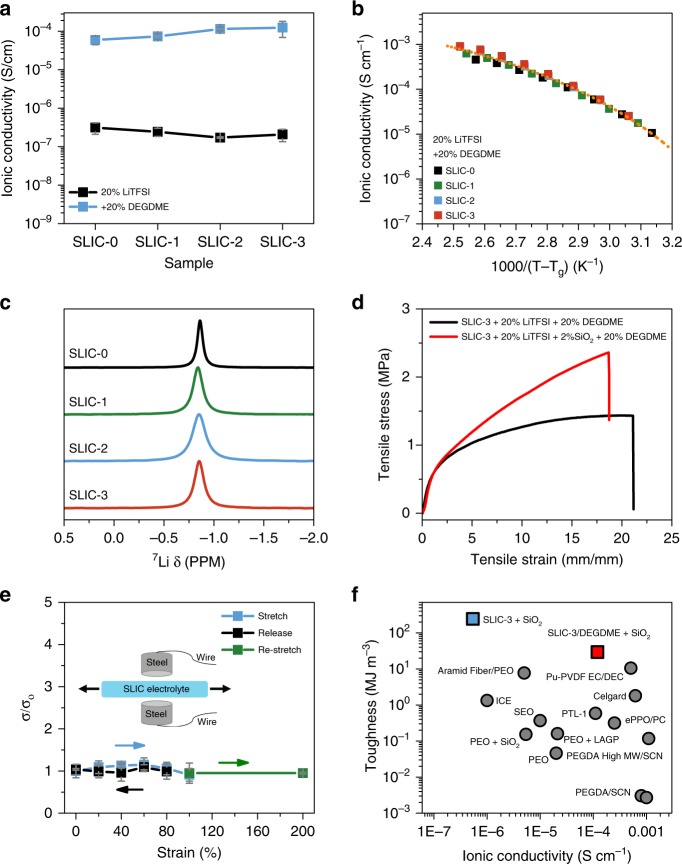
Characterization of SLIC as a polymer electrolyte. All samples contain 20 wt.% LiTFSI. **a** Ionic conductivity of plasticized and neat SLIC electrolytes as a function of UPy content in the polymer backbone. Measurement temperature is 25 °C. No SiO_2_ is present in these samples. **b** Ionic conductivity versus T_g_-shifted temperature for plasticized SLIC electrolytes with 20 wt.% LiTFSI, 20 wt.% DEGDME, and no SiO_2_. The orange dashed line serves to guide the eye. **c** ^7^Li NMR traces of the SLIC electrolytes dissolved in CDCl_3_ with 20 wt.% LiTFSI in each sample. **d** Stress-strain curves of plasticized SLIC-3 electrolytes with and without 2 wt.% SiO_2_. The strain rate is 100 mm/min **e** Normalized ionic conductivity as a function of strain for the SLIC electrolyte. This electrolyte incorporates SLIC-3 with 20 wt.% LiTFSI, 20 wt.% DEGDME, and 2 wt.% SiO_2_. Inset shows a schematic of the measurement apparatus **f** Comparison of the toughness and ionic conductivity of SLIC electrolytes to other electrolytes reported in literature. Details of comparison are included in the Supplementary Information.

One of the major advantages of the SLIC system for use as a polymer electrolyte is the decoupling of the ion conductivity from the mechanical properties of the polymer through the use of orthogonally functional H-bonding and ion-conducting domains. To investigate this decoupling, polymer electrolytes were created by dissolving lithium bis(trifluoromethanesulfonyl)imide (LiTFSI) into the polymer and casting a film. Ion-transport properties were investigated with and without the presence of diethylene glycol dimethyl ether (DEGDME) as a plasticizer. Experimentally, 20 wt.% LiTFSI and 20 wt.% DEGDME were chosen in the creation of polymer electrolyte films to maximize the ionic conductivity and mechanical properties of the samples (Supplementary Figs. 10–12). Figure 3a shows that the ionic conductivity for the SLIC polymer electrolytes remains relatively constant as the amount of hydrogen bonding, and thus the mechanical robustness, increases from SLIC-0 to SLIC-3. This observation is true for both the plasticized and unplasticized samples. Notably, the SLIC samples with 20 wt.% LiTFSI and 20 wt.% DEGDME have a high ionic conductivity value of around ~2 × 10^−4^ S cm^−1^ at room temperature. Table 1 shows the changes in maximum stress, strain, modulus, toughness, and Young’s modulus (E) of the SLIC samples as the amount of hydrogen bonding increases. It can be observed that while the toughness and modulus of the electrolytes increase two orders of magnitude from SLIC-0 to SLIC-3, the glass transition temperature and ionic conductivity of these samples remain nearly constant. These results highlight the ability of the supramolecular engineering strategy to decouple mechanical properties from ionic conductivity. This result stands in stark contrast to previous polymer electrolytes, where increases in mechanical strength resulted in decreases in ionic conductivity^36,41–43^.

**Table 1:** Mechanical properties of the plasticizer-free SLIC electrolytes with 20 wt.% LiTFSI along with the glass transition temperature (T_g_) and ionic conductivity (σ) of the samples at 70 °C. The young’s modulus is labeled as E.

Sample	Maximum stress (MPa)	Strain at break (mm mm^−1^)	Toughness (MJ m^−3^)	E (MPa)	T_g_ (°C)	σ_70 °C_ × 10^−5^(S cm^−1^)
SLIC-0	0.18 ± 0.02	126 ± 4.2	4.71 ± 0.32	0.17 ± 0.03	−29.6 ± 0.9	1.4 ± 0.23
SLIC-1	1.6 ± 0.15	215 ± 6.8	162 ± 1.3	0.87 ± 0.09	−28.2 ± 1.1	1.34 ± 0.15
SLIC-2	4.42 ± 0.27	66.3 ± 2.3	240 ± 2.2	3.45 ± 0.29	−29.5 ± 1.2	0.99 ± 0.06
SLIC-3	11.9 ± 0.96	34.6 ± 1.6	244 ± 3.1	5.20 ± 0.44	−29.9 ± 0.46	1.0 ± 0.12

The similarity between the ionic conductivities of the SLIC samples indicates that the soft PPG-PEG-PPG segment dictates the ionic conductivity, and that the conductivity is independent from the H-bonding UPy groups. We further investigated the ion-transport behavior to confirm this observation. Figure 3b shows that the T_g_ normalized temperature-dependent ionic conductivity of the SLIC electrolytes falls along a single master curve, indicating that the ion-transport mechanism is similar^44^. Furthermore, the VTF activation energies of all the SLIC samples are within 1 kJ mol^−1^ of one another, and the electrochemical impedance spectroscopy (EIS) traces are similar (Supplementary Figs. 13–15). A final piece of evidence that the soft segment dictates the conductivity of the SLIC samples comes from ^7^Li NMR measurements of SLICs dissolved in deuterated chloroform. ^7^Li NMR shows that the lithium solvation environment in the SLIC-LiTFSI and SLIC-LiTFSI-DEGDME complexes is relatively constant, regardless of the quantity of UPy in the backbone (Fig. 3c and Supplementary Fig. 16). This strongly suggests that the UPy groups do not interfere with the lithium solvation within the PPG-PEG-PPG backbone and that all the SLIC macromolecules solvate Li^+^ similarly^45–47^. These observations, in combination with the mechanical strength and ion conductivity measurements, demonstrate that the ion transport is indeed decoupled from the mechanical properties. With these data in hand, SLIC-3 was chosen to proceed with as a polymer electrolyte because of its extreme robustness and high ionic conductivity.

The effect of additives on the mechanical properties of the SLIC electrolytes is an important consideration for the final application of the stretchable polymer electrolytes. Addition of LiTSFI salt causes a slight decrease in the mechanical properties of the SLIC-based electrolytes (Supplementary Fig. 17). This is likely due to the large size of the TFSI anions interfering with chain packing and thus preventing aggregation of the UPy domains^48^. Indeed, the presence of salt causes a decrease in the 6 nm SAXS peak attributed to the UPy domains (Supplementary Fig. 18). In order to combat the negative effects of LiTFSI on the mechanical properties of the electrolyte, 2 wt.% SiO_2_ was added to the polymer electrolyte. Small amounts of ceramic additive are known to have several benefits including increasing the mechanical properties and enhancing the lithium transference number of polymer electrolytes^20^. Supplementary Fig. 19 show that while the SLIC-3 based electrolyte with 20% LiTFSI has good mechanical properties, the addition of 2 wt.% SiO_2_ can increase them further and also improve the elasticity of the samples. Finally, the effect of the plasticizer must be considered. When 20 wt.% DEGDME plasticizer is added, the mechanical properties of the electrolyte drop. However, as shown in Fig. 3d, addition of 2 wt.% SiO_2_ restores some of the original mechanical properties and the polymer remains elastic to over 100% strain (Supplementary Fig. 20). Even with the addition of LiTFSI and DEGDME, the SLIC-3 based polymer electrolyte is exceptionally robust. This electrolyte has a high ultimate stress of 2.5 ± 0.12 MPa and an extensibility of 1870 ± 110%. The SLIC-3 electrolyte with additives maintains a high degree of hydrogen bonding shown via FTIR (Supplementary Fig. 7) and has only minor creep at large strains (Supplementary Fig. 8). The effect of the addition of LiTFSI, DEGDME, and SiO_2_ on the ionic conductivity of the SLIC-3 based electrolytes is shown in Supplemental Fig. 21. The addition of 20 wt.% DEGDME results in a notable increase in ionic conductivity, while the addition of 2 wt.% SiO_2_ causes a modest decrease in the ionic conductivity that is well-documented^34^. The final choice for the high-performance polymer electrolyte is SLIC-3 with 20 wt.% LiTFSI, 20 wt.% DEGDME, and 2 wt.% SiO_2_. We confirmed that this electrolyte has no deleterious side reactions in a Li||SS electrochemical cell up to 4.0 V vs. Li/Li^+^ and has a respectable lithium transference number of 0.43 ± 0.04 (Supplementary Fig. 22). The rest of this paper will refer to the electrolyte with SLIC-3, 20 wt.% LiTFSI, 20 wt.% DEGDME and 2 wt.% SiO_2_ as the ‘SLIC electrolyte’.

Finally, when evaluating the performance of the SLIC electrolyte for a stretchable battery, the performance of the electrolyte under strain is a critical consideration. Figure 3e shows that the SLIC electrolyte can be stretched reversibly between 0 and 200% with very little change in the ionic conductivity. Supplementary Fig. 23 shows the corresponding EIS traces. We also demonstrate the versatility of the SLIC electrolyte system by showing that the SLIC electrolyte has high sodium ion conductivity when either NaTFSI or NaFSI are used in place of LiTFSI (Supplementary Fig. 24). The ability to conduct multiple types of ions will make the SLIC system useful for alternative battery chemistries.

Overall, the supramolecular design approach of the SLIC electrolyte combined with the judicious choice of electrolyte components makes this polymer electrolyte compelling for use in a stretchable battery. To confirm the uniqueness of this polymer, we compared SLIC to various other electrolytes reported in the literature (see Supplementary Fig. 25 and Supplementary Table 2 for details). The toughness of the polymer reflects how much energy a polymer can absorb upon deformation captures both strength and extensibility, and was therefore chosen as the most important metric to quantify the mechanical properties of a stretchable polymer electrolyte. It can be seen from Fig. 3f that the SLIC electrolyte has a record-high toughness of 29.3 ± 1.4 MJ m^−3^, which is at least three-fold higher than the most robust electrolytes reported to date. Even compared to non-ionically conducting state-of-the-art dynamic elastomers, the SLIC-3 electrolyte demonstrates competitive toughness and extensibility (Supplementary Fig. 9). Furthermore, the ionic conductivity of the SLIC-3 electrolyte with 20 wt.% LiTFSI, 20 wt.% DEGMDE, and 2 wt.% SiO_2_ reaches 1.2 ± 0.21 × 10^−4^ S cm^−1^, which competes with the highest reported ionic conductivities. This high ionic conductivity is acceptable for use in lithium-ion battery applications. Achievement of such high ionic conductivity and toughness is enabled by using supramolecular engineering to enhance the mechanical strength of the polymer via hydrogen bonding without compromising ionic conductivity.

### SLIC as a stretchable electrode binder material

**Fig. 4:**
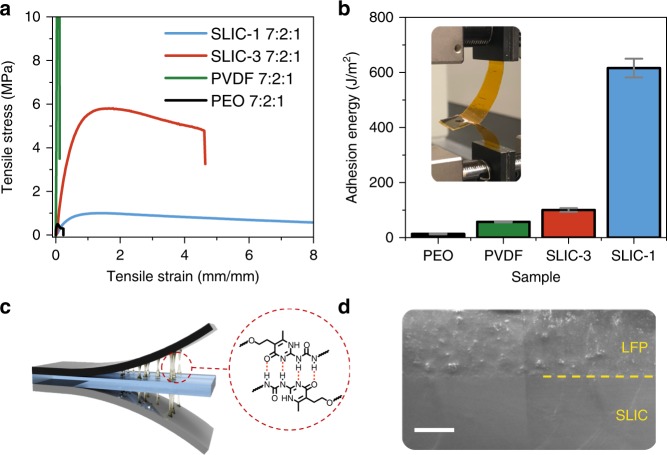
Use of SLIC to construct stretchable electrode materials. **a** stress-strain curves of polymer composite electrodes with different amounts of LFP and carbon black. The ratios given are in terms of Polymer:LFP:CB. No other components (DEGDME/SiO_2_) are included. The strain rate is 100 mm/min. **b** Adhesion energy between the SLIC electrolyte and various composite electrodes with composite ratio of 7:2:1. Inset shows an optical image of the measurement setup. **c** Schematic showing the formation of dynamic UPy bonds at the electrode-electrolyte interface. **d** SEM image of the interface between SLIC-3 electrolyte and a 7:2:1 SLIC-1 based electrode. Scale bar is 25 µm.

In addition to functioning as an excellent polymer electrolyte, SLIC is an attractive material for use as a stretchable electrode binder. Previous attempts to create stretchable battery electrodes either utilize cost-intensive micro/nanoscale engineering^24,26,49^, or involve coating a small amount of active material onto an elastic support^50–52^. Ideally, intrinsically stretchable electrodes could be fabricated by replacing the stiff polymer binder in conventional electrodes with a stretchable one. Because SLIC is a polymer with excellent mechanical properties as well as ion conductivity, it is an obvious candidate for making stretchable composite electrode materials. By using a conventional slurry process, we were able to create large-scale, free standing electrodes based on mixtures of lithium iron phosphate (LiFePO_4_, LFP), carbon black (CB), and SLIC electrolyte (Supplemental Fig. 26). Note that for the electrodes, the SLIC electrolyte contains 20 wt.% LiTFSI but does not include plasticizer or ceramic additive. Unless otherwise specified, electrode compositions are given as the mass ratio of polymer:LFP:CB. Figure 4a shows the unique advantage ability of the ultra-tough SLIC polymers to create stretchable electrodes compared to conventional polymer binders such as PVDF and PEO. At a ratio of 7:2:1, the SLIC-1 and SLIC-3 based electrodes can be stretched to an impressive 940 ± 61% and 450 ± 48%, respectively. PVDF and PEO based electrodes, on the other hand, can only be stretched to around 20% strain. Supplemental Fig. 27 show the effects of electrode composition on the mechanical properties of stretchable composite electrodes made with SLIC-1 and SLIC-3, respectively. Generally, as the composition of active material increases, the stiffness of the composite increases and the extensibility decreases. It can be seen that while the SLIC-3 electrodes have higher modulus and strength, the extensibility is roughly half that of the SLIC-1 electrodes. The higher extensibility of the SLIC-1 based electrodes is attributed to the ability of the softer polymer to accommodate more stiffening from the addition of rigid active materials. Notably, SLIC-1 electrodes achieve an extensibility of nearly 100% at a ratio of 2:7:1, showing that the electrodes maintain good stretchability even when a high loading of 70 wt.% of LFP active material is used. Achievement of 100% strain of the electrode component with a mass loading of 70 wt.% LFP surpasses the 50–65 wt.% loading achieved in previous stretchable electrode works utilizing elastic binders^53^. Using an elastic binder is also superior to commonly used methods of spray/dip-coating rigid electrodes onto stretchable substrates^51,54^. These spray/dip-coating methods ultimately limit the overall energy density of the final electrode^55^.

One challenge for batteries with a solid or gel electrolyte is achieving good interfacial contact and ion conductivity at the electrode/electrolyte interface^4^. Because of the dynamic nature of the UPy bonds, it is expected that the SLIC electrodes will be able to form strong interfaces with the SLIC electrolyte. Figure 4b shows the results of interfacial adhesion tests between the SLIC electrolyte and composite electrodes (7:2:1) containing SLIC-1, SLIC-3, PEO, and PVDF. Raw data for the adhesion test are shown in Supplementary Figure 28. It can be seen that the adhesion energy between the SLIC electrodes and the SLIC electrolyte is much greater than for electrodes made from conventional polymers. The notable increase in adhesion energy for the SLIC electrodes is attributed to hydrogen-bonding at the interface, illustrated schematically in Fig. 4c. The electrode with SLIC-1 has particularly high adhesion energy, which can be attributed to the fact that the SLIC-1 polymer is more flowable than the SLIC-3 polymer, as evidenced by the rheometry in Fig. 1d. This flowability allows the SLIC-1 polymer to have more adhesion through increased Van der Waals interactions and at the same time form more adhesive H-bonds between the electrode and the electrolyte^30^. The SEM image in Fig. 4d shows that the interface between the SLIC-1 based electrode and the SLIC electrolyte is indeed seamless and continuous. We also confirmed that the SLIC-based electrodes have higher adhesion energy to current collectors than either PEO or PVDF electrodes (Supplemental Fig. 29). The combination of the impressive extensibility of the SLIC-based composite electrodes and the ability to form well-adhered interfaces makes the SLIC material promising for use as a stretchable supramolecular battery material.

### Stretchable batteries

The properties discussed thus far demonstrate the distinct advantages of SLIC compared to previously reported polymeric ion conductors. The dynamic crosslinking of UPy units in SLIC allows for a unique decoupling of mechanical properties and ionic conductivity, which is not commonly observed in polymer electrolytes. This allows for the creation of a polymer electrolyte with record-high toughness and high ionic conductivity. Furthermore, the mechanical and ion-transport properties of SLIC allow it to be used for an effective electrode binder material, surpassing the active material loading and stretchability achieved based on previously reported elastomeric binders. The unique nature of the dynamic UPy bonds also allows for the formation of intimately bonded electrode-electrolyte interfaces, which is a phenomenon that has not been previously reported. These promising properties demonstrate that SLIC should serve as an excellent candidate for an ion-transport polymer in deformable battery systems. In the final portion of this manuscript, we will provide a brief demonstration of a high-performance stretchable LIB based on SLIC materials.

Battery testing on these stretchable electrode and electrolyte materials was first conducted in coin cells in order to determine their performance in a conventional LIB. Supplementary Fig. 30 shows the long-term cycling at a rate of C/5 of a SLIC-1 composite electrode (7:2:1) paired with the SLIC electrolyte and a lithium counter electrode. The SLIC-based battery can cycle at a rate of C/5 for over 400 cycles with an average coulombic efficiency of 99.45% and capacity retention of 86.8%. Furthermore, the battery achieves rate capability of up to 1 C at room temperature (Supplementary Fig. 31). Cyclic voltammetry of the battery from 2.5 to 3.8 V shown in Supplemental Fig. 32 shows that no side reaction or degradation happens in these battery materials over the voltage range of interest. Overall, SLIC-based battery materials can function with excellent performance in conventional lithium ion batteries and have no obvious deleterious effects.

**Fig. 5:**
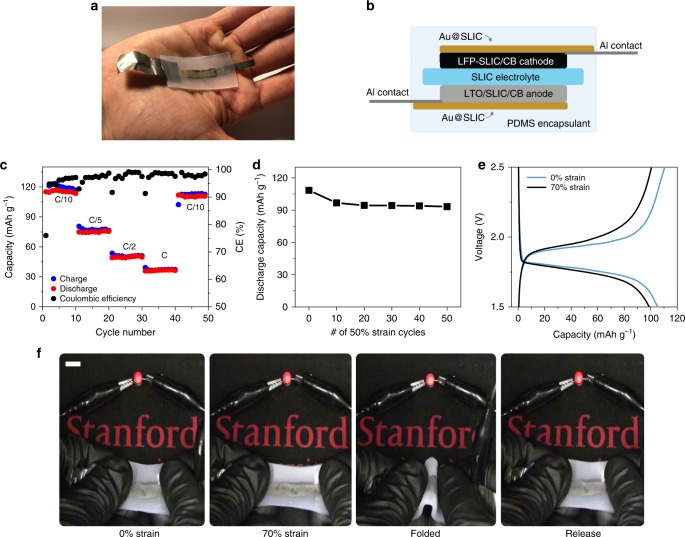
Stretchable batteries based on SLIC. **a** Optical photograph of the conformable battery based on all SLIC components. **b** Cross-sectional view of the SLIC battery showing the layout of the components. **c** Capacity versus cycle number for a full-cell based on stretchable SLIC components. The active material loading in the electrodes (LFP or LTO) is 70 wt.%, ~1.1 mAh cm^−2^. **d** Discharge capacity of an all-SLIC stretchable battery after several cycles of 50% strain. Discharge capacity is measured after each set of 10 stretching cycles. **e** Performance of an all-SLIC stretchable battery under 0 and 70% strain. **f** Demonstration of a stretchable SLIC battery providing power to a red LED under no strain, stretched 70%, folded, and returned to its original position.

As a final demonstration, we provide initial evidence that SLIC, a novel ion-conducting electrolyte and binder, can be used to fabricate an all-stretchable battery. Figure 5a shows an optical photograph of a deformable battery that utilizes stretchable electrodes, electrolyte, and current collectors based on SLIC polymers. The entire stack is encapsulated in PDMS. Figure 5b shows a cross-sectional schematic of the battery layout. Creation of this battery requires the development of a SLIC-based stretchable current collector. To create this current collector, the conventional method of microcracked gold was utilized because of its high conductivity and electrochemical stability^56–58^. A thin layer of gold (~100 nm) was thermally evaporated onto a SLIC substrate. The SLIC electrode slurry was then cast directly onto the gold current collector. The Au@SLIC current collector has low resistance of 20 Ω/□ that remains relatively constant even upon stretching to 100% strain (Supplementary Fig. 33). SEM was used to confirm the formation of the gold microcrack structure on the current collector upon stretching (Supplementary Fig. 34). Even with the electrode coating, the Au@SLIC current collector can be stretched elastically to over 300% of its initial length (Supplementary Fig. 33).

To demonstrate the ability to fabricate full cells based on the stretchable SLIC electrode and electrolyte components, a lithium titanate (Li_4_Ti_5_O_12_, LTO) anode was fabricated in the same manner as the LFP electrode. Figure 5c shows the rate capability of a full-cell containing LFP||SLIC-3||LTO. Note that for the full-cell, SLIC-1 electrodes with a ratio of 2:7:1 were used. These electrodes have 70% active material (LFP or LTO), resulting in a high mass loading of about 1.1 mAh cm^−2^. This areal capacity is high compared to most stretchable batteries, and is similar to commercially available flexible batteries (Supplementary Tables 3–4). The areal capacity of our stretchable battery is not significantly reduced from conventional electrode materials, which range from ~2–4 mAh cm^−2^ ^59^. The full-cell batteries based on the stretchable SLIC components can obtain impressive capacities of nearly 120 mAh g^−1^ with coulombic efficiencies reaching over 99%. Supplementary Fig. 35 shows the charge-discharge traces of the full cell at a rate of C/10 at cycle 1 and cycle 45, indicating that these stretchable materials last for many cycles in a full cell configuration. Traces of the battery operating at different rates are shown in Supplementary Fig. 35.

The ability of the all-SLIC battery to handle deformation during operation is a key indicator of its eventual application in flexible/stretchable electronics. We first performed charge-discharge cycles of the full cells with intermittent stretching at 50% strain between cycles. Figure 5d shows that the capacity of the SLIC-battery drops from 108 to 97 mAh g^−1^ after the first set of 10 stretching cycles. The corresponding charge-discharge traces are shown in Supplemental Fig. 36. After the first set of 10 cycles, the capacity stabilizes and decreases only 4% over the next 40 stretching cycles. Furthermore, SEM analysis shows that the electrode-electrolyte layers remain well-adhered and crack-free throughout the rigorous 50% stretching cycles (Supplemental Fig. 37). This indicates that SLIC may function to help alleviate the well-documented delamination problem of stretchable electronics^60^. We attribute the slight decrease in capacity upon stretching to the minor increase in resistance of the Au@SLIC current collectors due to the formation of microcracks as discussed previously. We also confirmed the ability of the SLIC-based battery to operate during deformation conditions. The PDMS-encapsulated full cells were operated both unstretched and with 70% strain applied (Fig. 5e). It can be observed that there is a marginal decrease in capacity from 108 mAh g^−1^ to 99 mAh g^−1^ and a slight increase in overpotential in response to the 70% applied strain. This slight decrease in capacity is likely also due to increased resistance of the Au@SLIC current collector. Finally, as a demonstration, the stretchable SLIC-based battery was charged and used to power a red LED (Supplementary Movie 1). The red LED remains lit even when the SLIC battery is stretched up to 70% strain, and folded in half (Fig. 5f).

The performance of the SLIC-based battery is compared to previous reported stretchable batteries in Supplementary Table 3. It is important to note that the majority of the previously reported stretchable batteries rely on strain-engineering approaches including creating interconnected rigid islands^23^, buckled electrodes^24,26^, rod-like structures^61–65^, microstructured electrodes^51,52,66,67^, and origami structures^25,68^. While all of these strategies delivered promising results, the expensive micro/nano fabrication procedures used to create such batteries are tedious and potentially cost-prohibitive. On the contrary, our SLIC-based system is able to deliver competitive performance while using intrinsically stretchable components that can be prepared with facile solution casting processes. Indeed, the fabrication of SLIC-based stretchable battery components is similar to the slurry-casting processes used in conventional battery manufacturing^69^. We also note the advantage of the use of a robust and tough polymer electrolyte for a stretchable battery, which is a major advantage compared to the majority of stretchable battery electrolytes that rely on a flowable or liquid electrolyte^70,71^ (Supplementary Table 3).

The SLIC-based battery is one of first demonstrations of an intrinsically stretchable lithium ion battery. The use of an ultra-tough elastomer as an electrolyte and binder as opposed to strain-engineering techniques may enable scalable stretchable batteries with improved energy densities. More detailed battery testing is currently limited by the availability of effective water-impermeable stretchable packaging material. An improved current collector may also enable SLIC-based batteries to function at even higher strains. Further work improving the performance of SLIC batteries is underway. Nevertheless, the performance of the SLIC-based battery highlights the ability of the unique polymer system to create all-stretchable battery materials that function in an intrinsically stretchable lithium ion battery.

## Discussion

In conclusion, the supramolecular lithium ion conductor, SLIC, is a rationally designed, macromolecule that enables the fabrication of high-performance ion-conducting materials for stretchable LIBs. SLIC’s dynamically crosslinked network design incorporates orthogonally functional components that provide both high ionic conductivity and excellent toughness. Using this design to decouple ionic conductivity and mechanical robustness, we fabricated a polymer electrolyte that is tougher than any reported previously. Additionally, the ultra-robust and ionically conductive nature of the SLIC polymers lends them to work as excellent binder materials to create stretchable composite electrodes using a traditional slurry-casting process. Combining these stretchable materials allows for the creation of an intrinsically stretchable lithium ion battery based purely on SLIC materials. The SLIC system demonstrated here provides an exciting avenue to create tough ion-conducting materials for future studies involving conformable and stretchable electrochemical devices.

## Methods

### Synthesis of SLIC materials

All reagents were commercially available and used as supplied without further purification. Poly(propylene glycol)-*block*-poly(ethylene glycol)-*block*-poly(propylene glycol) (PPG-PEG-PPG, *M*_n_ = 2000), isophorone diisocyanate (IPDI), 1,5-pentanediol (PD) (Sigma-Aldrich, USA) was dried under vacuum at 80 °C overnight before use. Dibutyltin dilaurate (DBTDL) catalyst was purchased from Alfa Aesar. The prepolymer^72^ and 5-(2-hydroxyethyl)-6-methyl-2-aminouracil **2**^73^ were prepared according to published procedures. ^1^H NMR spectra were recorded on a Varian Mercury 400 NMR spectrometer at room temperature with use of the deuterated solvent as the lock and the residual solvent or TMS as the internal reference^7^. Li NMR spectra were collected using an Inova 300 MHz spectrometer. Gel permeation chromatography (GPC) was carried out in DMF on two PolyPore columns (Agilent) connected in series with a DAWN multiangle laser light scattering (MALLS) detector and an Optilab TrEX differential refractometer (both from Wyatt Technology).

### Physical characterization of SLIC materials

DSC experiments were carried out with a TA Instruments DSC Q2000 using Tzero Aluminum pans. Mechanical tensile-stress and adhesion strength experiments were performed using an Instron 5848 Microtester with a 10 N force transducer. Adhesion measurements were carried out using a Instron 5848 Microtester with a 10 N force transducer. Adhesion energies were calculated using a previously reported method^74^. Creep measurements were performed using a TA DMA Q800. Rheological experiments were carried out using a stress-controlled rheometer (TA Instruments Model AR-G2) with an 8 mm parallel plate attachment. The small-angle X-ray scattering (SAXS) measurements on polymer films in transmission geometry were carried out on beamline 4–2 at Stanford Synchrotron Radiation Lightsource (SSRL) of SLAC National Accelerator Laboratory (SLAC). Field emission scanning electron microscope (FESEM) (JEOL JSM-7600F) was employed to observe the surface morphology under 5 kV gun voltage. FTIR spectra were measured using a Nicolet iS50 FT/IR Spectrometer (Thermo Fisher) with a diamond attenuated total reflectance (ATR) attachment.

### Fabrication of SLIC electrolytes

SLIC polymers (0.54 g) were dissolved in 11 mL of THF along with an appropriate amount of vacuum-dried LiTFSI and 14 nm fumed SiO_2_. After dispersing, the viscous solution was degassed and cast into a Teflon mold, and dried for 24 h at RT. After drying at RT, the film was further dried for 24 h at 60 °C in a vacuum oven and for 24 h in a nitrogen-filled glovebox. Resulting films were 20–200 µm thick. The electrolytes were plasticized by addition of a defined amount of DEGMDE with a micropipette. The electrolytes were then allowed to swell with the added plasticizer for 1 h prior to use.

### Fabrication of SLIC electrodes

SLIC polymer (250 mg) and LiTFSI (63 mg) were dissolved in N-methyl-2-pyrrolidone (500 uL) to make a viscous liquid. Active material (LFP/LTO, MTI), and carbon black (Timcal SuperP) were then added in appropriate weight ratios ranging from 7:2:1 polymer:LFPLCB to 2:7:1. The amount of carbon black remained fixed at 10 wt.%. The slurries were mixed using a dual asymmetric centrifugal mixer (FlackTek). Resulting slurries were doctor bladed onto either a Teflon block or current collector and then dried for 12 h at RT and 24 h at 70 °C under vacuum. Films were rapidly transferred into a nitrogen-filled glovebox, peeled, and then cut to the appropriate size.

### Electrochemical characterization

All electrochemical measurements were performed using a Biologic VSP-300 potentiostat. Temperature controlled experiments utilized a Espec environmental chamber. Electrochemical impedance measurements were conducted by sandwiching polymer films in a symmetric stainless steel (SS||SS) coin cell. A Teflon spacer of 150 µm was used to ensure no thickness change during the measurement. For all electrochemical tests, samples were transferred hermetically to an argon filled glovebox. Non-stretchable battery tests were conducted using an Arbin battery cycler in 2032 coin cells. A 2 cm^2^ disk of plasticized electrolyte was placed on top of a freshly scraped 1 cm^2^ Li disk. A 1 cm^2^ composite electrode coated onto an Al current collector was placed on top of the electrolyte and the stack was sealed in the coin cell.

### Fabrication of stretchable batteries

Stretchable current collectors were fabricated by thermal evaporation of a gold film of 40–100 nm onto a thin (20 µm) film of SLIC-3. The evaporation rate was 8 Å s^−1^. The strain-dependence of electronic resistance was measured using a customized stretcher and resistance monitor (Agilent E4980A precision LCR Meter). To make stretchable batteries, the composite electrode slurry was doctor-bladed with a gap height of 10–200 µm directly onto the Au@SLIC film. Following drying in the vacuum glovebox at 60 °C, Au@SLIC + electrode slurries were transferred into the nitrogen-filled glovebox. In the glovebox, the SLIC electrolyte was plasticized, and the components were assembled in the following order: Au@SLIC + LTO || SLIC electrolyte || Au@SLIC + LFP. Aluminum tabs were taped to the edge of the Au@SLIC current collectors, and the entire stack was sandwiched between two slabs of PDMS (EcoFlex DragonSkin 10 Medium) and sealed with a coating of liquid PDMS. Typical stretchable batteries had an active material area of 1 cm^2^.

## Supplementary information

Supplementary Information

Description of Additional Supplementary Files

Supplementary Movie 1

## Data Availability

The authors declare that the main data supporting the findings of this study are available within the article and its Supplementary Information files. Extra data are available from the corresponding author upon reasonable request.
