# Supplementary material for: Decoupling of mechanical properties and ionic conductivity in supramolecular lithium ion conductors
Source: Nat Commun. 2019 Nov 26;10:5384. doi: 10.1038/s41467-019-13362-4 (PMC6879760; doi:10.1038/s41467-019-13362-4)
Supplement: Supplementary file 1 — Supplementary Information [file 41467_2019_13362_MOESM1_ESM.pdf]

Supplementary Information

Decoupling of Mechanical Properties and Ionic Conductivity in Supramolecular Lithium Ion Conductors

Mackanic et al.

Supplementary Figures

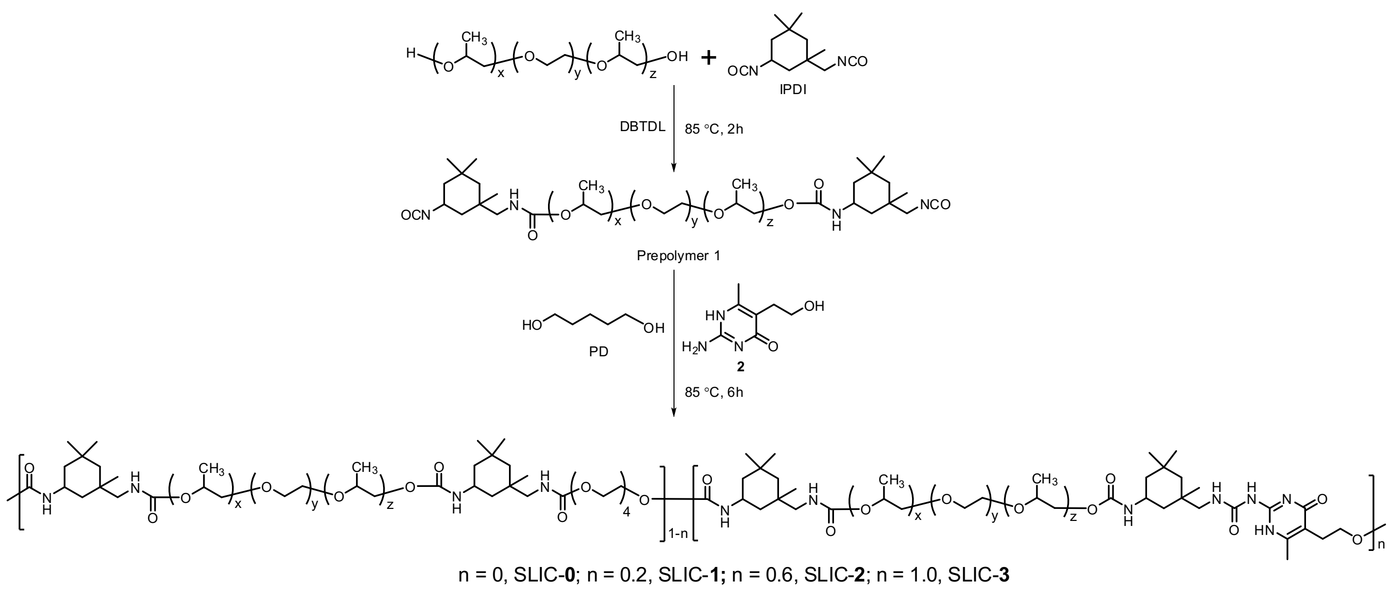

Supplementary Figure 1. Synthetic scheme of SLIC polymers.

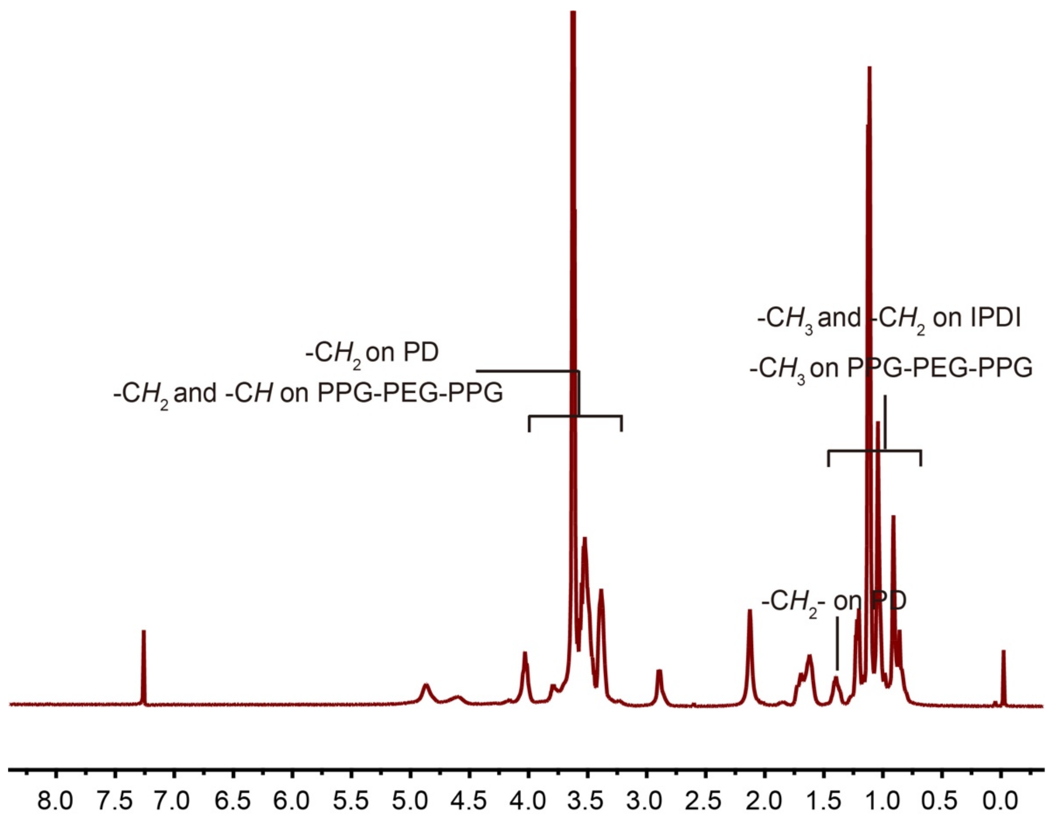

Supplementary Figure 2. 1H NMR of SLIC-0. ($CDCl_3$, room temperature, 400 MHz)

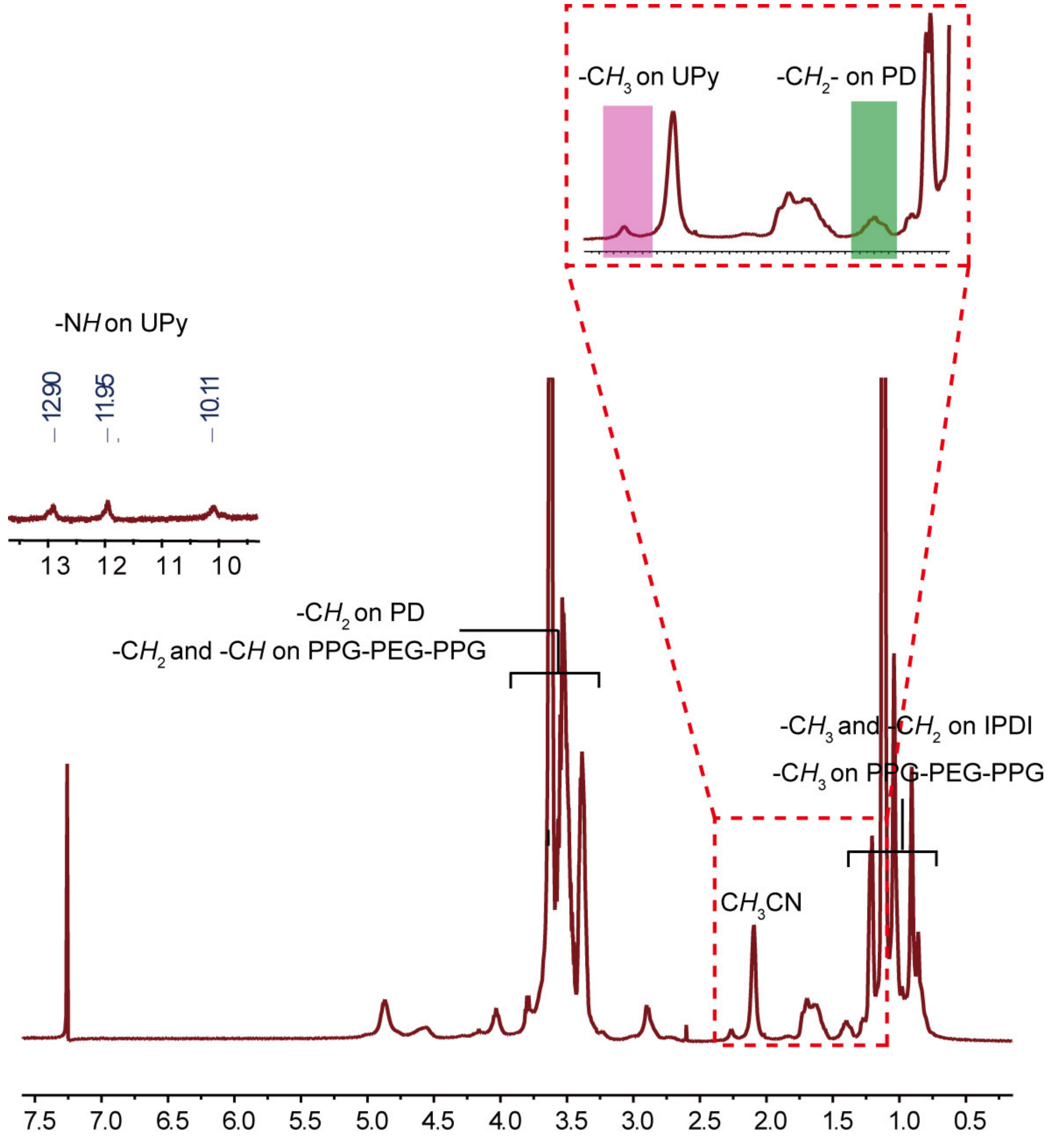

Supplementary Figure 3. 1H NMR of SLIC-1. ($CDCl_3$, room temperature, 400 MHz)

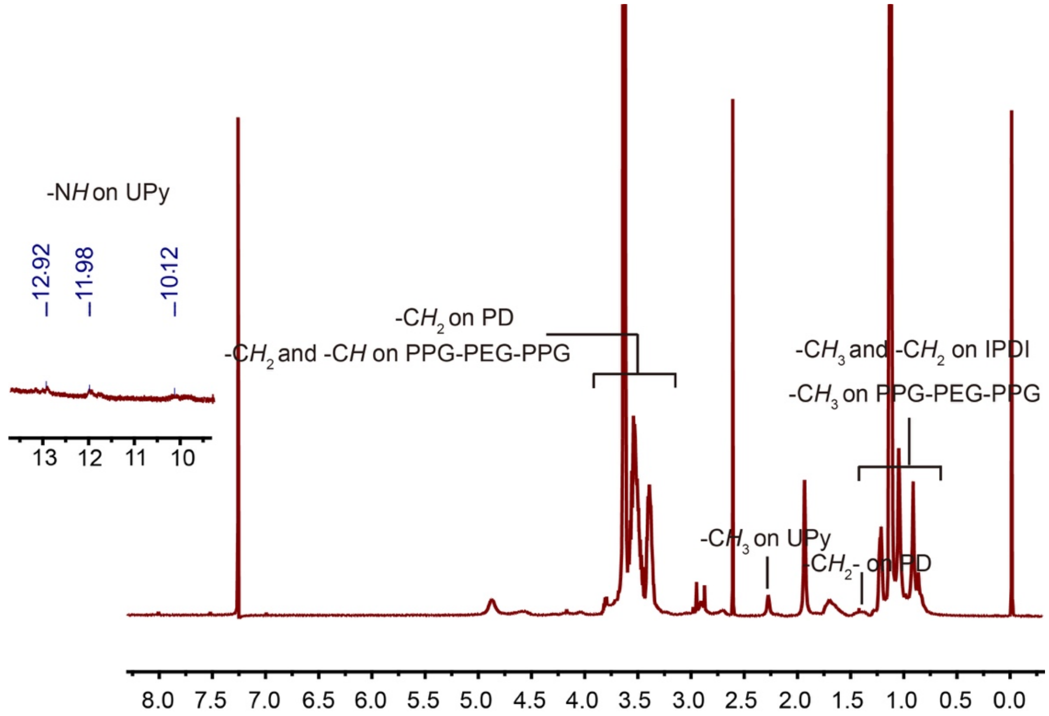

Supplementary Figure 4. 1H NMR of SLIC-2. ($CDCl_3$, room temperature, 400 MHz)

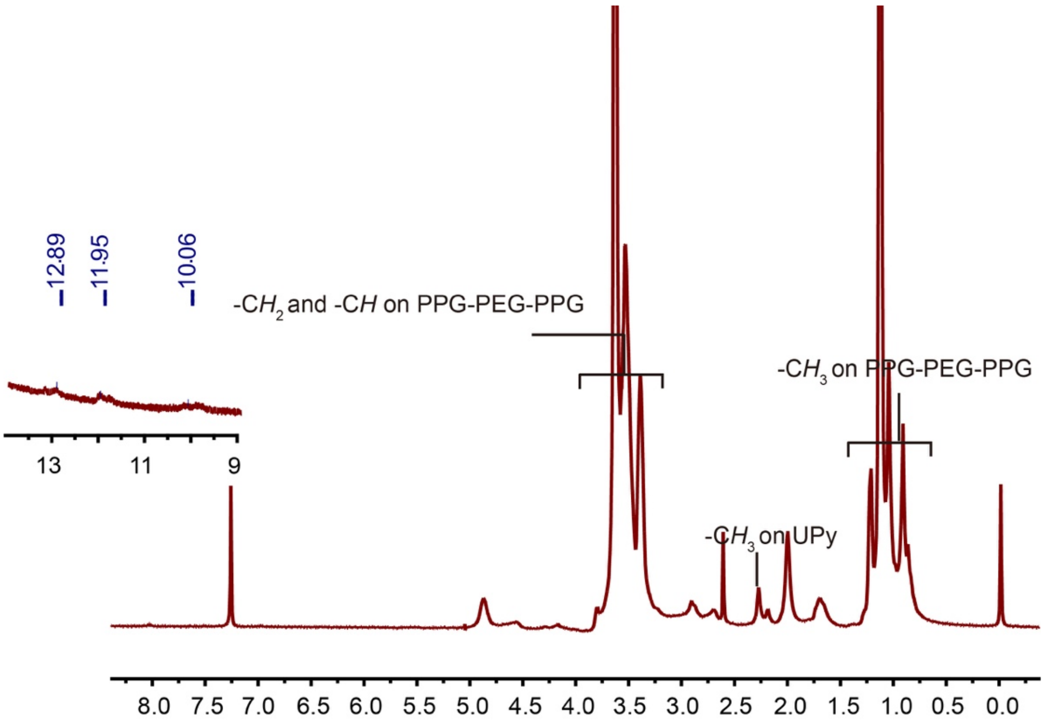

Supplementary Figure 5. 1H NMR of SLIC-3. ($CDCl_3$, room temperature, 400 MHz)

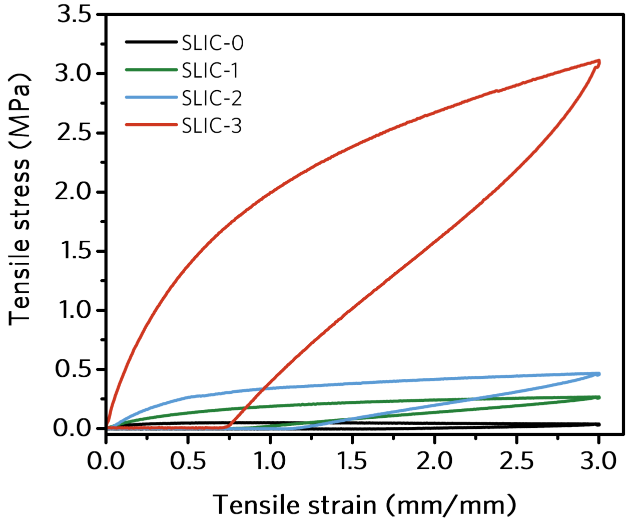

Supplementary Figure 6. Strain cycling of SLIC Polymers. The strain hysteresis is less for polymers containing more hydrogen bonding groups. The strain rate is 30 mm min^{-1}.

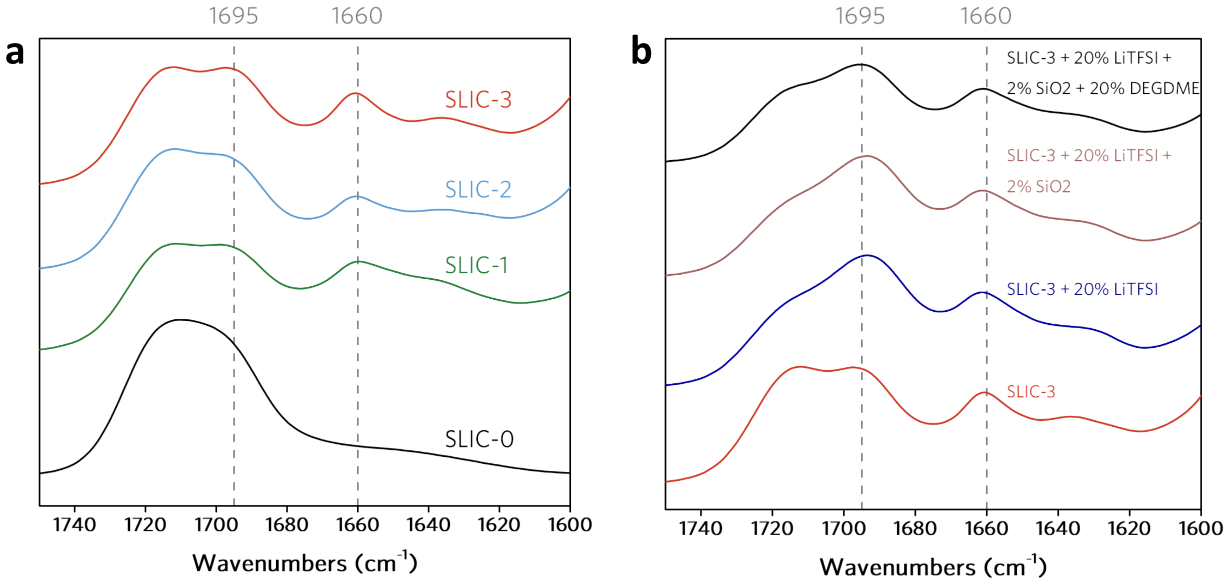

Supplementary Figure 7. FTIR Characterization of SLIC electrolytes. **a** FTIR Spectra from SLIC-0 to SLIC-3 in the range of 1600-1750 cm^{-1}. The peak associated with H-bonded C=O in urea (~1660 cm^{-1}) increases from SLIC-0 to SLIC-3.[1] Similarly, the peak associated with H-bonded C=O in urethane (~1695 cm^{-1}) increases as the amount of UPy in the SLIC polymer increases. The increases in these peaks shows the clear increase in the amount of hydrogen bonding present in the polymers as the amount of UPy increases. **b** FTIR Spectra from SLIC-3 electrolytes with additives. It can be seen that the addition of LiTFSI, SiO_2, and DEGDME have minimal effect on the hydrogen bonding of the SLIC polymers.

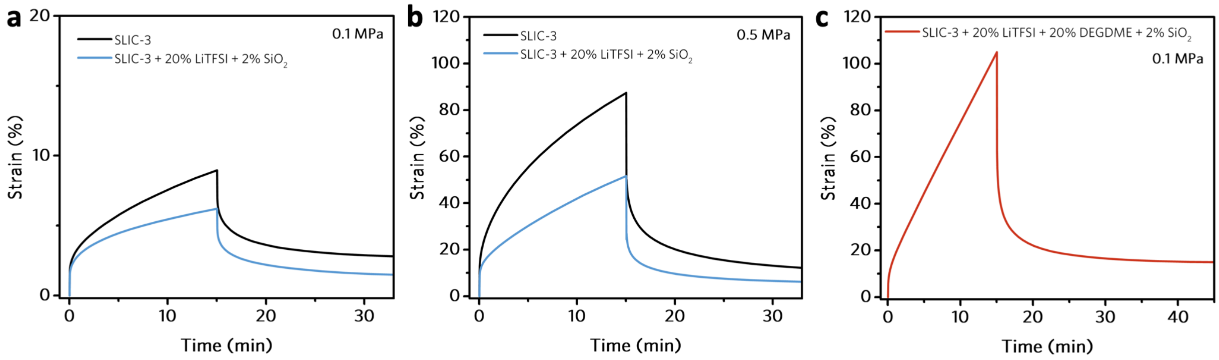

Supplementary Figure 8. Creep behavior of the SLIC polymer and the SLIC electrolytes. A fixed step stress was applied for 15 minutes and then relaxed. **a** SLIC-3 and SLIC-3 + 20% LiTFSI + 2% SiO_2 with a step stress of 0.1 MPa applied. **b** SLIC-3 and SLIC-3 + 20% LiTFSI + 2% SiO_2 with a step stress of 0.5 MPa applied. **c** SLIC-3 + 20% LiTFSI + 20% DEGDME + 2% SiO_2 with a step stress of 0.1 MPa applied. In all cases, the viscoelastic SLIC molecules demonstrate good strain recovery.

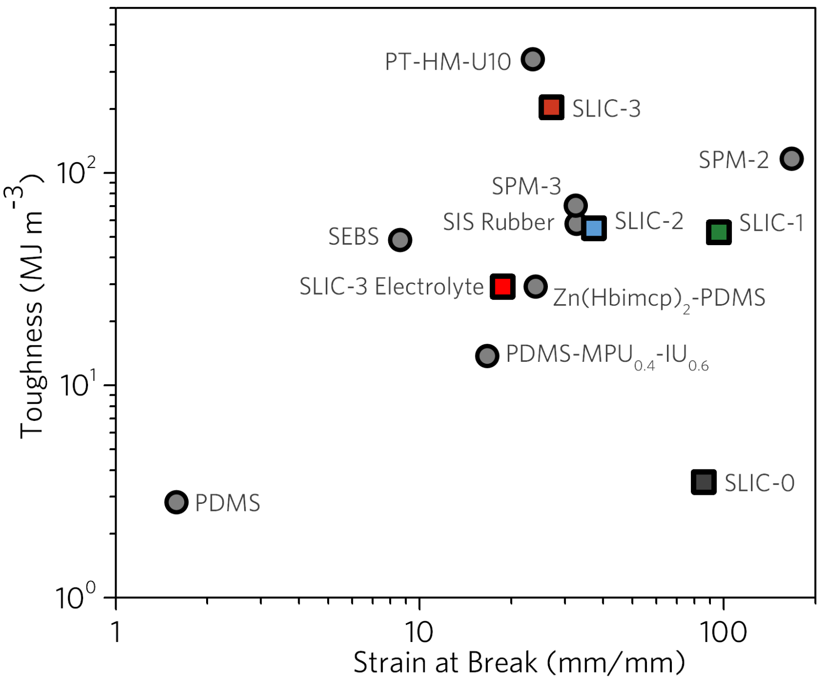

Supplementary Figure 9. Comparison of SLIC polymers to other commercial polymers and recently reported tough macromolecules. The Y-axis is the toughness of the polymers and the X-axis is the strain at break for the polymers. These properties are important for stretchable applications because it is important to both have very stretchable polymers as well as materials that can absorb a lot of energy before breaking. SLIC-0 through SLIC-3 are represented on the graph. It can be seen that the SLIC polymers are among the toughest reported dynamic macromolecules. Specifically, SLIC-3 has very high toughness. All of the SLIC polymers except for the non-dynamic SLIC-0 have a very good combination of high toughness and extensibility. Furthermore, even when the SLIC-3 electrolyte with 20 wt.% LiTFSI, 20 wt.% DEGDME, and 2 wt.% SiO2 is compared to state-of-the-art elastomers, it demonstrates a good combination of toughness and stretchability.

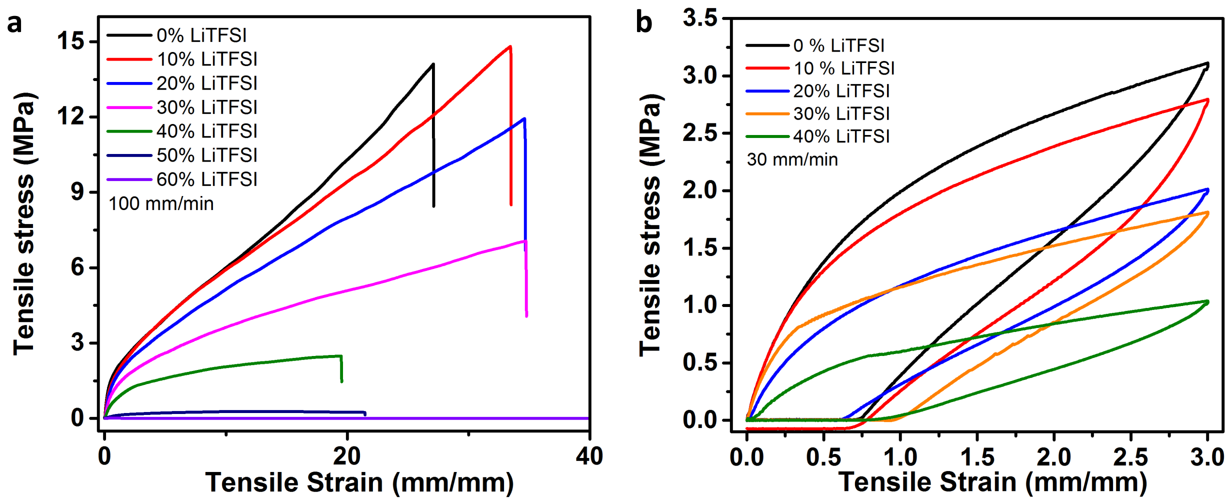

Supplementary Figure 10. Mechanical properties of SLIC-3 as a function of LiTFSI concentration. **a** Stress strain curves for SLIC-3 based electrolyte with different amounts of lithium loading. Mechanical properties remain intact until around 40% LiTFSI, after which there is a drastic reduction in strength. **b** Cyclic stress-strain curves for SLIC-3 with different LiTFSI loadings. Samples were cycled continuously between 0 and 300% strain at a rate of 30 mm/min.

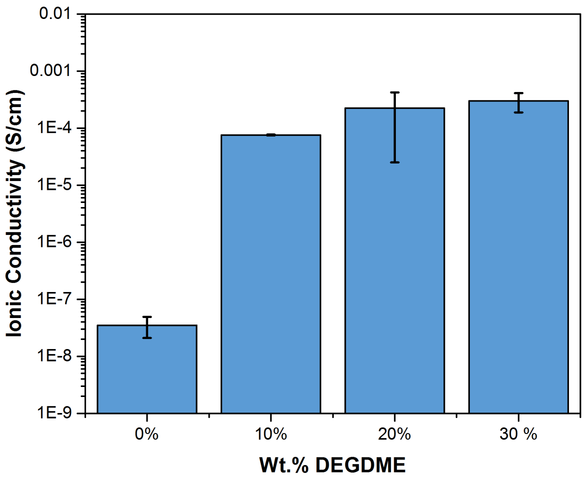

Supplementary Figure 11. Ionic conductivity of SLIC-3 as a function of DEGDME content. The salt loading is 20% LiTFSI. It can be seen that after 20% DEGDME is added, additional DEGDME does not drastically increase the ionic conductivity.

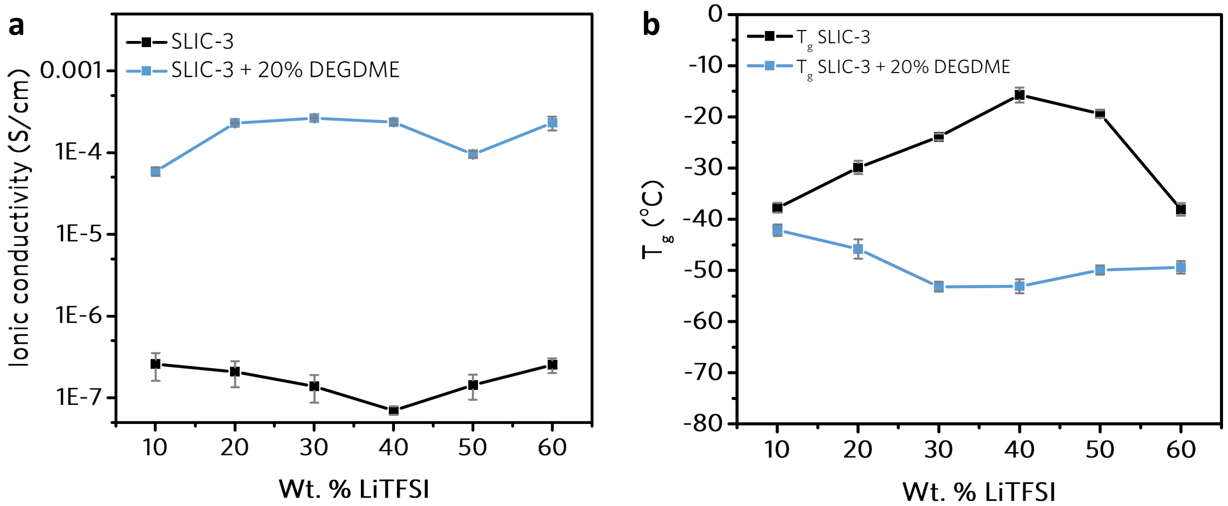

Supplementary Figure 12. Ion conductivity and glass transition as a function of LiTFSI Concentration for SLIC-3. **a** Ionic conductivity of SLIC-3 based electrolytes as a function of LiTFSI concentration with and without 20% DEGDME plasticizer. Measurement temperature is 25 °C. **b** Glass transition temperature of SLIC-3 based electrolytes as a function of LiTFSI concentration with and without 20% DEGDME plasticizer. For both samples, the measured ionic conductivity correlates with changes in the glass transition temperature. Initially, increased salt causes increased ionic crosslinking up until 40% LiTFSI, increasing the T_g and lowering ionic conductivity. Above 40% LiTFSI, the presence of large amounts of the bulky TFSI hinders chain packing, leading to a lowered T_g and enhanced ionic conductivity. This correlates well to the sharp drop in mechanical properties that is observed above 40 wt.% LiTFSI. For the plasticized samples, these effects are less obvious because the primary mechanism for ionic conductivity is through partially solvated Li^+

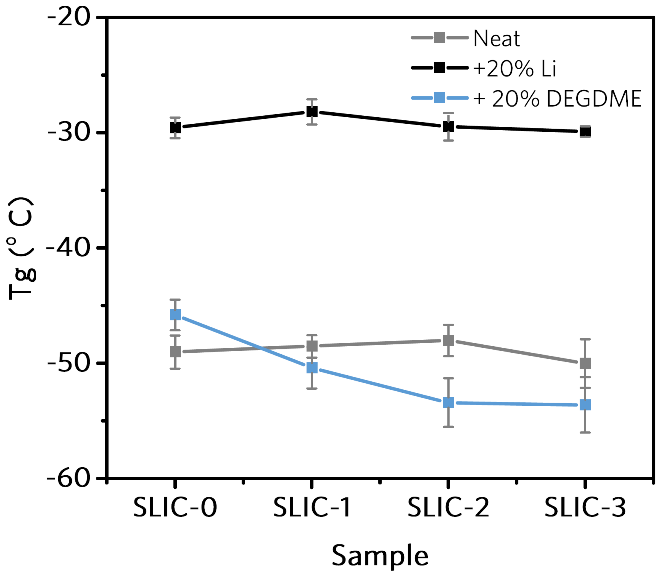

Supplementary Figure 13. Glass transition temperature of various SLIC samples. T_g of the different SLIC films with 20% LiTFSI and plasticizer. The addition of 20% LiTFSI causes a drastic increase in the T_g, which is then lowered by the addition of the DEGDME plasticizer. As UPy concentration increases, the T_g of the plasticized sample becomes progressively lower. This is potentially caused by interaction of the UPy groups with the DEGDME.

a

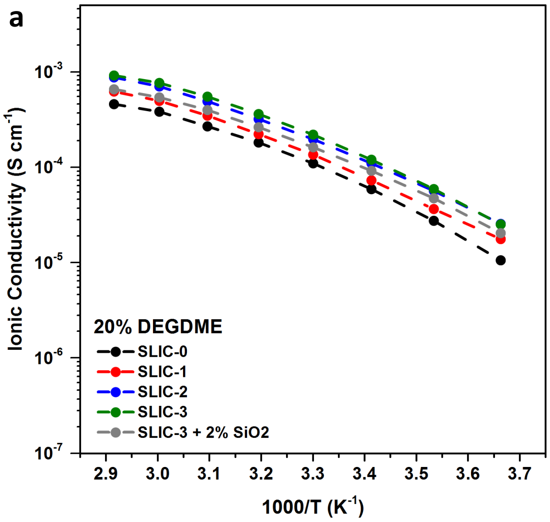

b

$$\sigma(T) = \frac{A}{T^{0.5}} * Exp\left(-\frac{B}{R\left(T-(T_g-50)\right)}\right)$$

c

Sample	A (S $K^{0.5}$ cm^{-1})	E_a (kJ mol^{-1})	R^2
SLIC-0	0.90	6.38	0.992
SLIC-1	1.89	7.18	0.996
SLIC-2	2.71	7.34	0.996
SLIC-3	2.32	7.03	0.991

Supplementary Figure 14. Temperature-dependent ionic conductivity and activation energy of SLIC-3 electrolyte. **a** Temperature dependent ionic conductivity of SLIC samples with 20 wt.% LiTFSI and 20 % DEGDME. **b** Vogel-Tamman-Fulcher Equation. **c** VTF activation energies (B) and exponential prefactors (A) extracted using the Vogel-Tamman-Fulcher equation. The T_gs for each sample were used in the calculation.

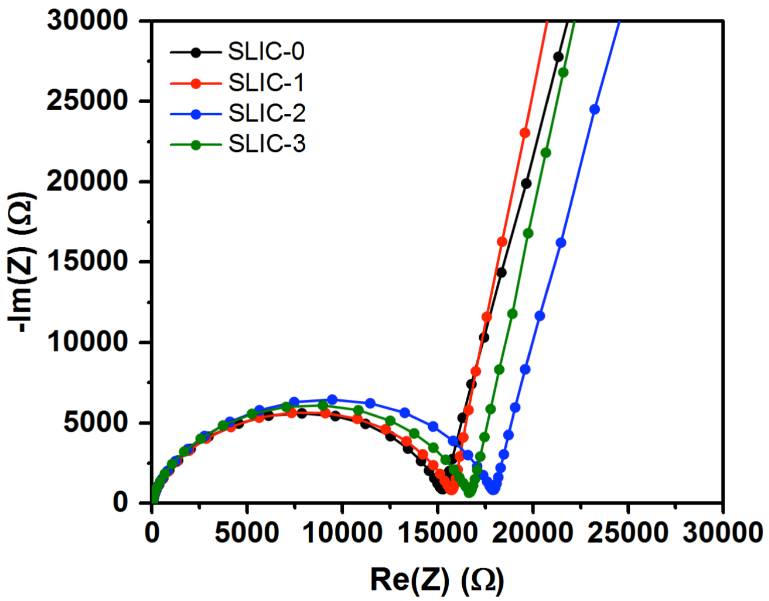

Supplementary Figure 15. EIS Traces of SLICs at room temperature.

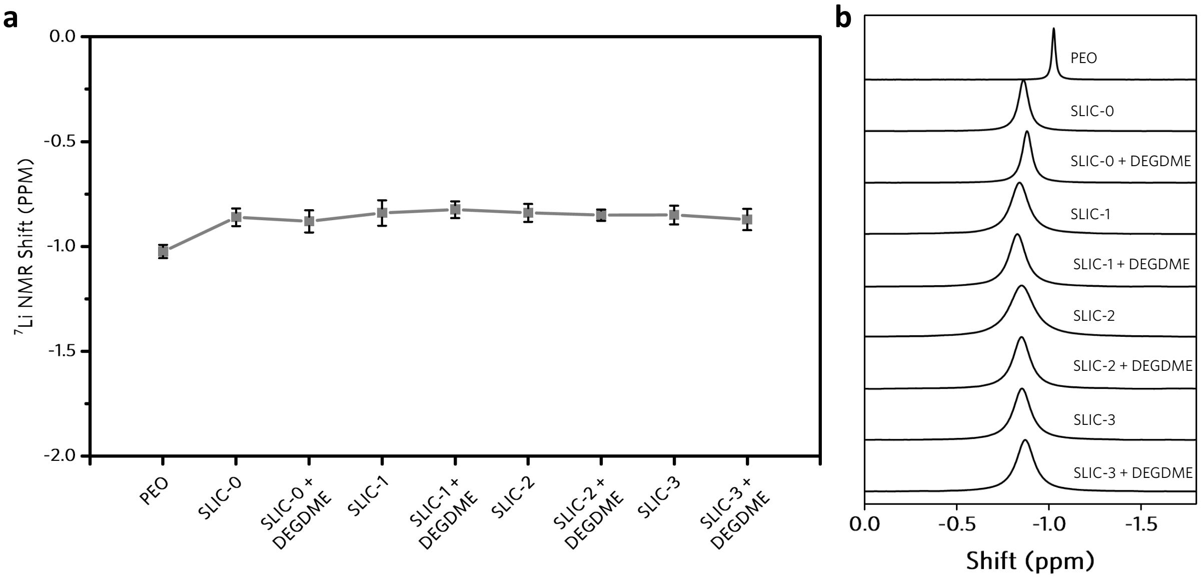

Supplementary Figure 16. ^{7}Li NMR of SLIC electrolytes dissolved in deuterated chloroform. All samples contain 20 wt.% LiTFSI. Plasticized samples contain an additional 20 wt.% DEGDME. All experiments are carried out in deuterated chloroform, which does not solvate LiTFSI and thus should not affect the coordination environment. The lithium coordination environment does not change drastically for any of the SLIC samples.

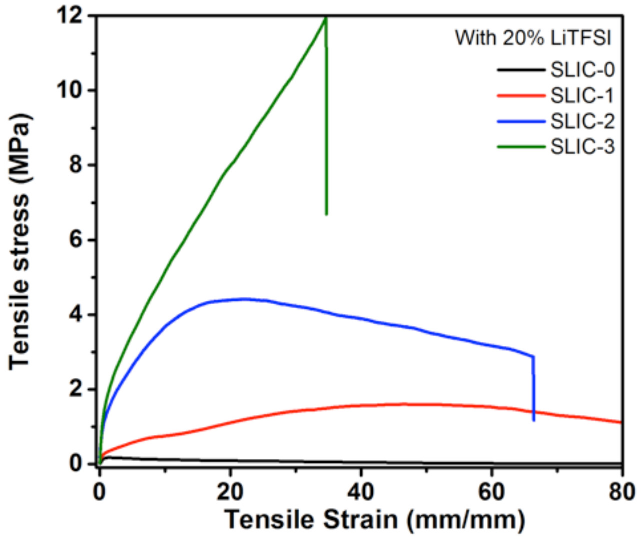

Supplementary Figure 17. Stress-Strain measurements of SLIC-0 to 3 with 20% LiTFSI. The mechanical properties are slightly decreased compared to the salt-free polymer films.

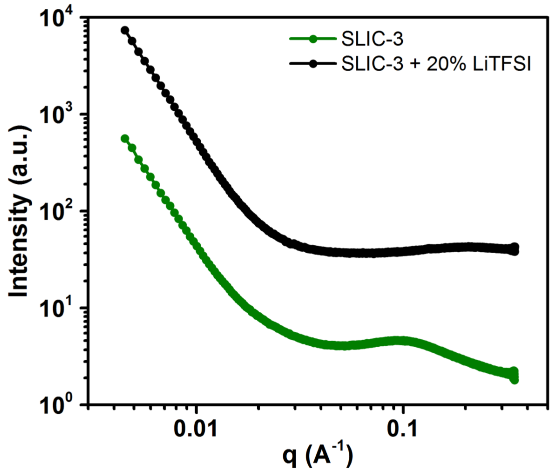

Supplementary Figure 18. SAXS of SLIC-3 with and without LiTFSI. The decrease in the 6 nm SAXS peak suggests that the bulky TFSI group of LiTFSI is interfering with the ordering of the polymer, reducing the formation of aggregated UPy crosslinks.

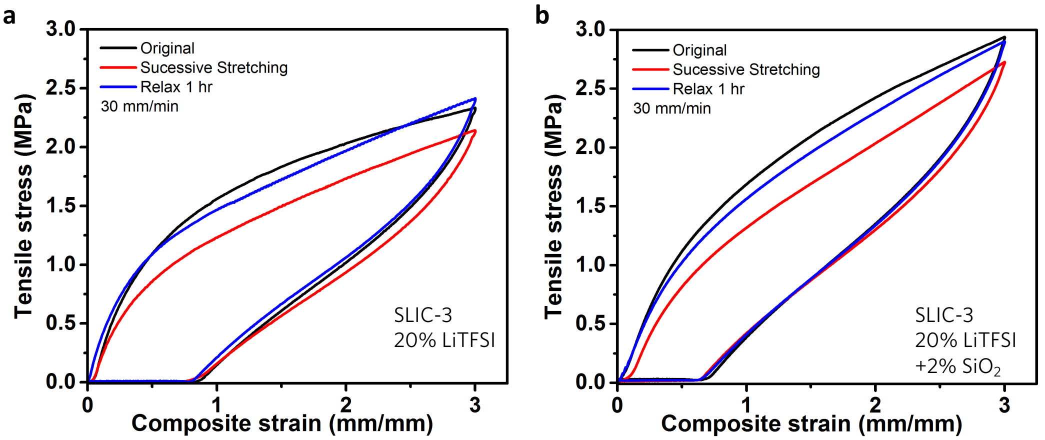

Supplementary Figure 19. Cyclic stress-strain curves of SLIC-3 with 20% LiTFSI and 2 wt.% SiO_2. **a** SLIC-3 + 20 wt. % LiTFSI. The elasticity is retained in the presence of LiTFSI. **b** SLIC-3 + 20 wt.% LiTFSI + 2 wt.% SiO_2. By including 2 wt.% SiO_2, the mechanical properties are retained and there is improved strain cycling.

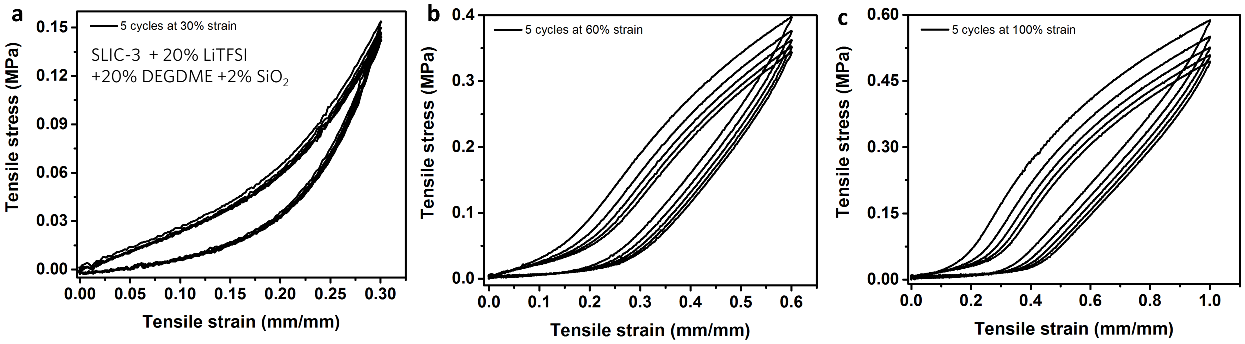

Supplementary Figure 20. Cyclic Strain of Plasticized SLIC-3 Electrolytes. The electrolyte contains 20% LiTFSI, 20% DEGDME, and 2% SiO_2. The samples were strained cyclically at a rate of 30 mm/min to **(a)** 30%, (**b**) 60%, and **(c)** 100% strain.

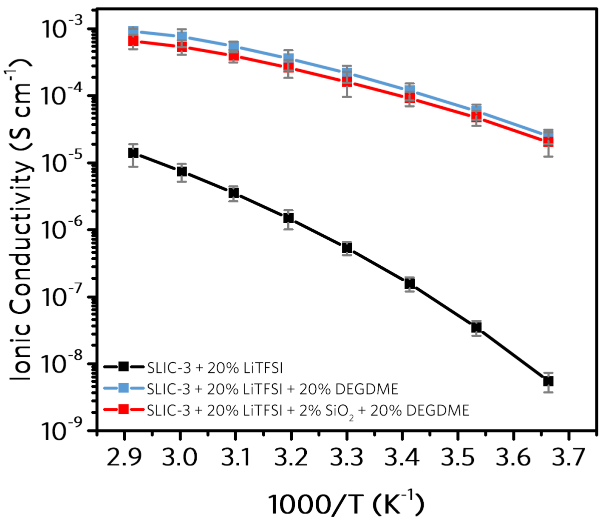

Supplementary Figure 21. Effect of additives on temperature-dependent ionic conductivity of SLIC-3 electrolyte. Addition of plasticizer causes a dramatic increase in the ionic conductivity, adding SiO_2 causes a slight decrease.

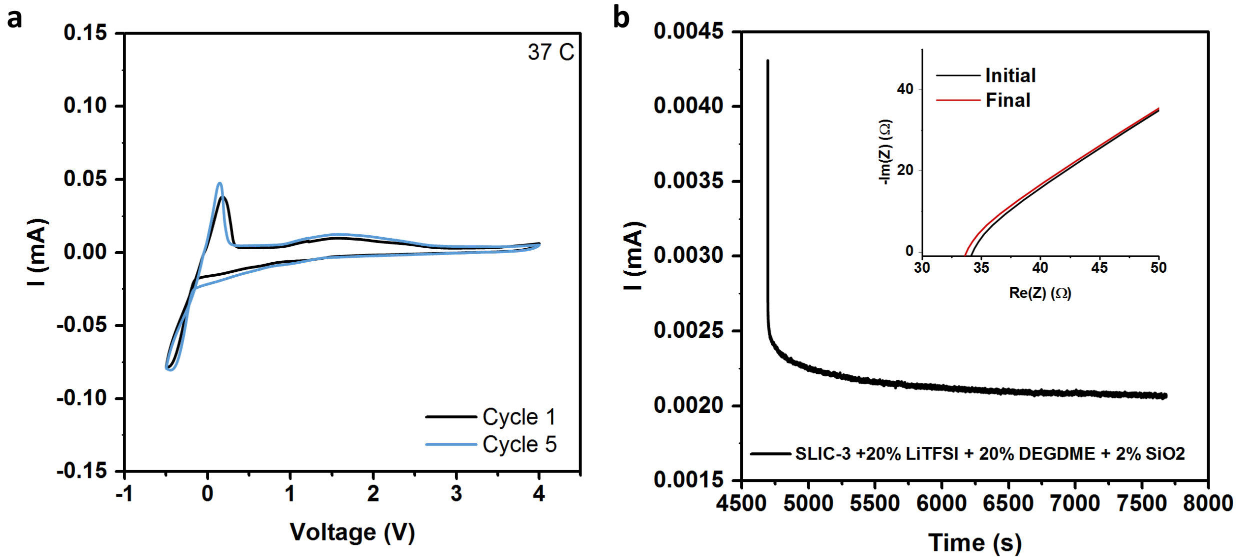

Supplementary Figure 22. Electrochemical characterization of the SLIC-3 electrolyte. Samples contain 20% LiTFSI, 20% DEGDME, and 2% SiO_2. Measurements were carried out at 37 °C to minimize effects of thermal fluctuation. **a** Electrochemical stability measured in a SS||SLIC-3||Li configuration. The scan rate is 0.1 mV s^{-1}. **b** Transference number measurement in a Li||Li symmetric cell. The measurement was made using the method of potentiostatic polarization.[2] In this experiment, a fixed voltage of 50 mV is applied and the decay in current is observed. impedance is measured before and after polarization to correct for polarization resistance. The calculated transference number is 0.43.

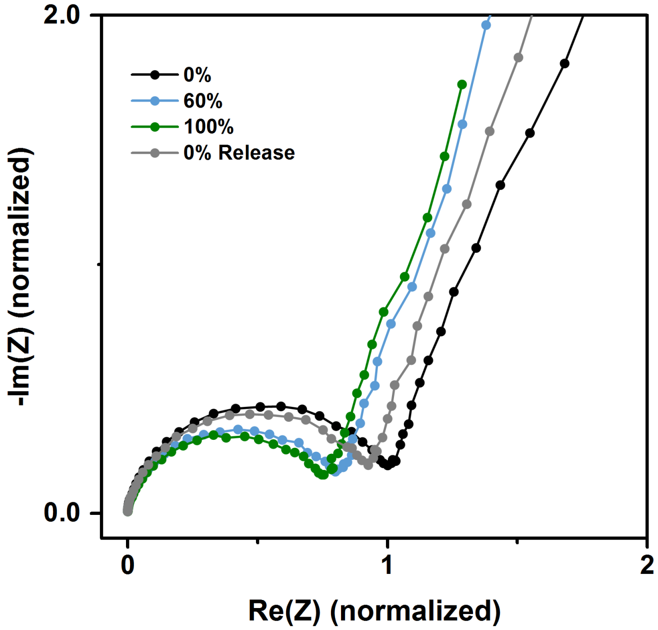

Supplementary Figure 23. EIS Spectra as a function of strain. A SLIC-3 electrolyte is used. The ionic conductivity is normalized to the resistance of the unstrained sample. The slight decrease in conductivity observed is due to the sample thickness becoming thinner as the stretching increases. To report the final normalized ionic conductivity, the change in thickness was measured and accounted for. In this experiment, the SLIC-3 electrolyte with 20% LiTFSI, 20% DEGDME, and 2% SiO_2 was fixed to an extension stage in a air-free environment. Two steel plates were placed on either side of the stretched sample and attached externally to a potentiostat. The sample was deformed by a fixed amount, and then the EIS trace was measured.

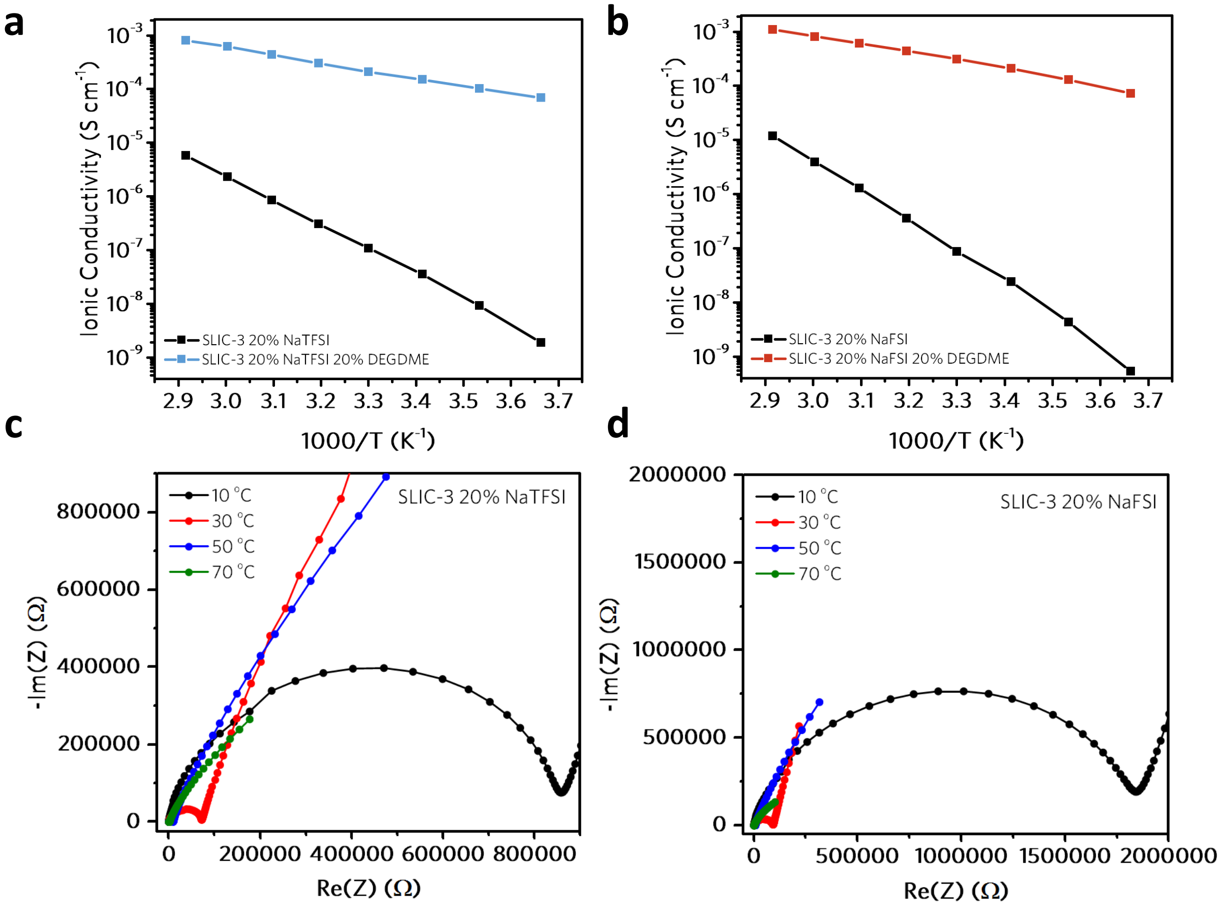

Supplementary Figure 24. Demonstration of the ability of the SLIC electrolyte to conduct multiple ion species. **a** Conductivity versus temperature graphs for SLIC-3 electrolytes with 20% NaTFSI (black) and with 20% NaTFSI and 20% DEGDME (blue). **b** Conductivity versus temperature graphs for SLIC-3 electrolytes with 20% NaFSI (black) and with 20% NaFSI and 20% DEGDME (red). **c** EIS traces for the SLIC-3 electrolyte with 20% NaTFSI at 10, 30, 50, and 70 °C. **d** EIS traces for the SLIC-3 electrolyte with 20% NaFSI at 10, 30, 50, and 70 °C. It can be seen that the sodium ion conductivity of the SLIC electrolyte is similar to the lithium ion conductivity. Specifically, at 30 °C SLIC-3 electrolytes with NaTFSI and NaFSI have ionic conductivities of $2.1*10^{-4}$ and $3.2*10^{-4}$, respectively.

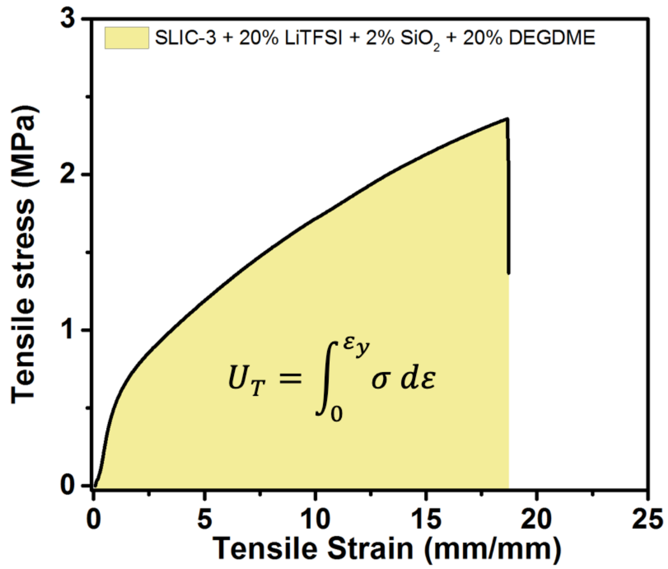

Supplementary Figure 25. Calculation of toughness. The integrated area under the curve in order to calculate the toughness of SLIC-3 with 20% LiTFSI , 2% SiO2, and 20% DEGDME.

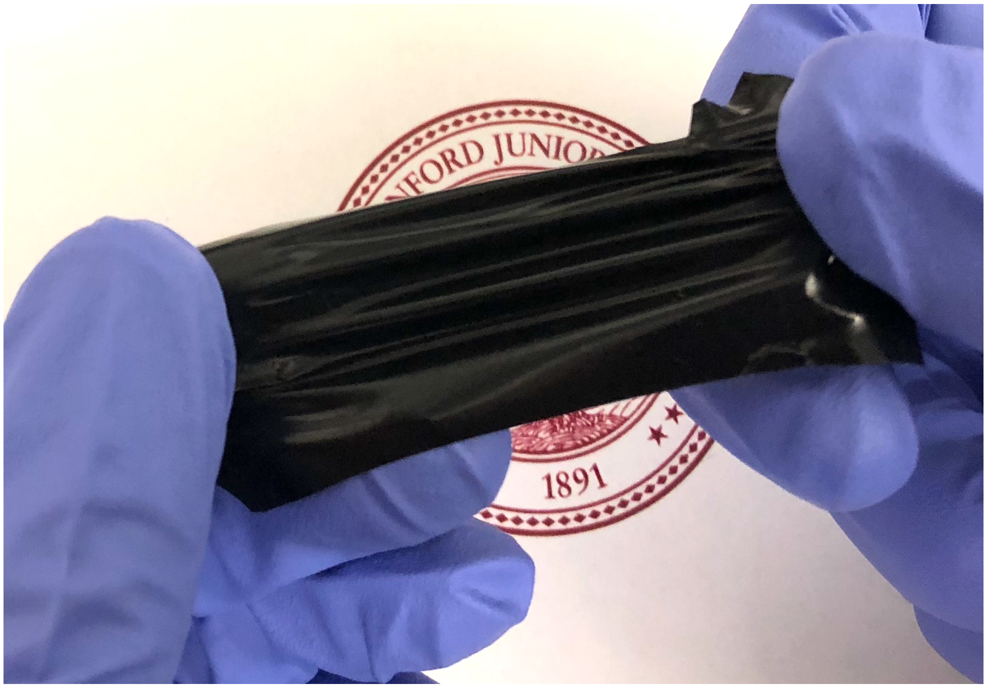

Supplementary Figure 26. Photograph of free-standing composite electrode. Composition of the electrode in the picture is 70 wt.% SLIC-1, 20 wt.% LFP, 10 wt.% Carbon Black.

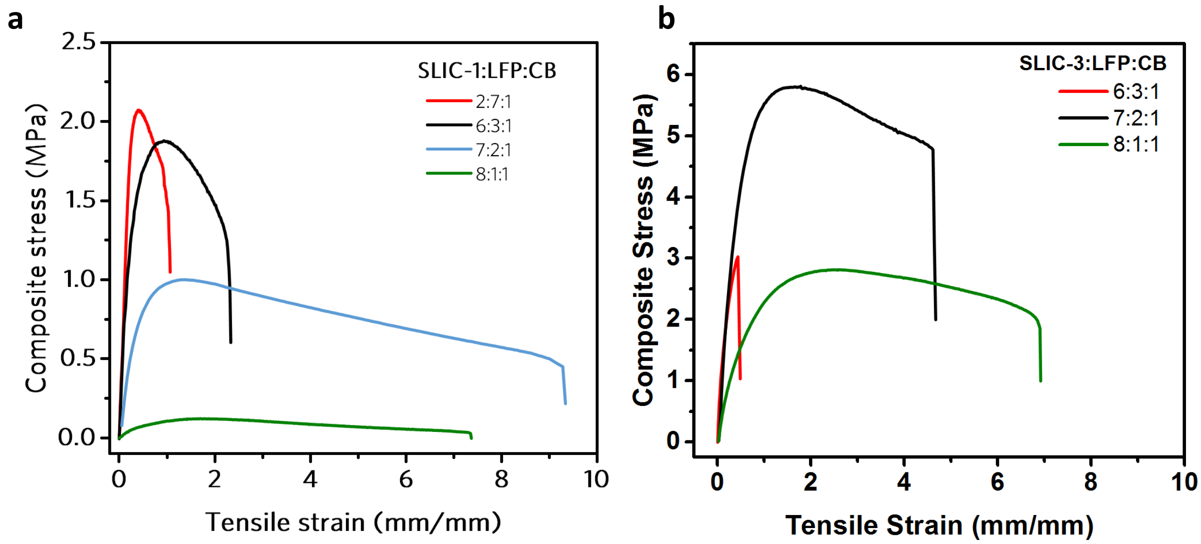

Supplementary Figure 27. Mechanical properties of composite electrodes as a function of electrode composition. The ratios are given as the ratio of polymer:LFP:CB. **a** Using SLIC-1 as a binder **(b)** Using SLIC-3 as a binder. It can be seen that in general, increasing the amount of the polymer increases the stretchability of the electrode. SLIC-1 creates more extensible electrodes than SLIC-3 because the softer polymer binder is able to accommodate more stiffening from the addition of active materials.

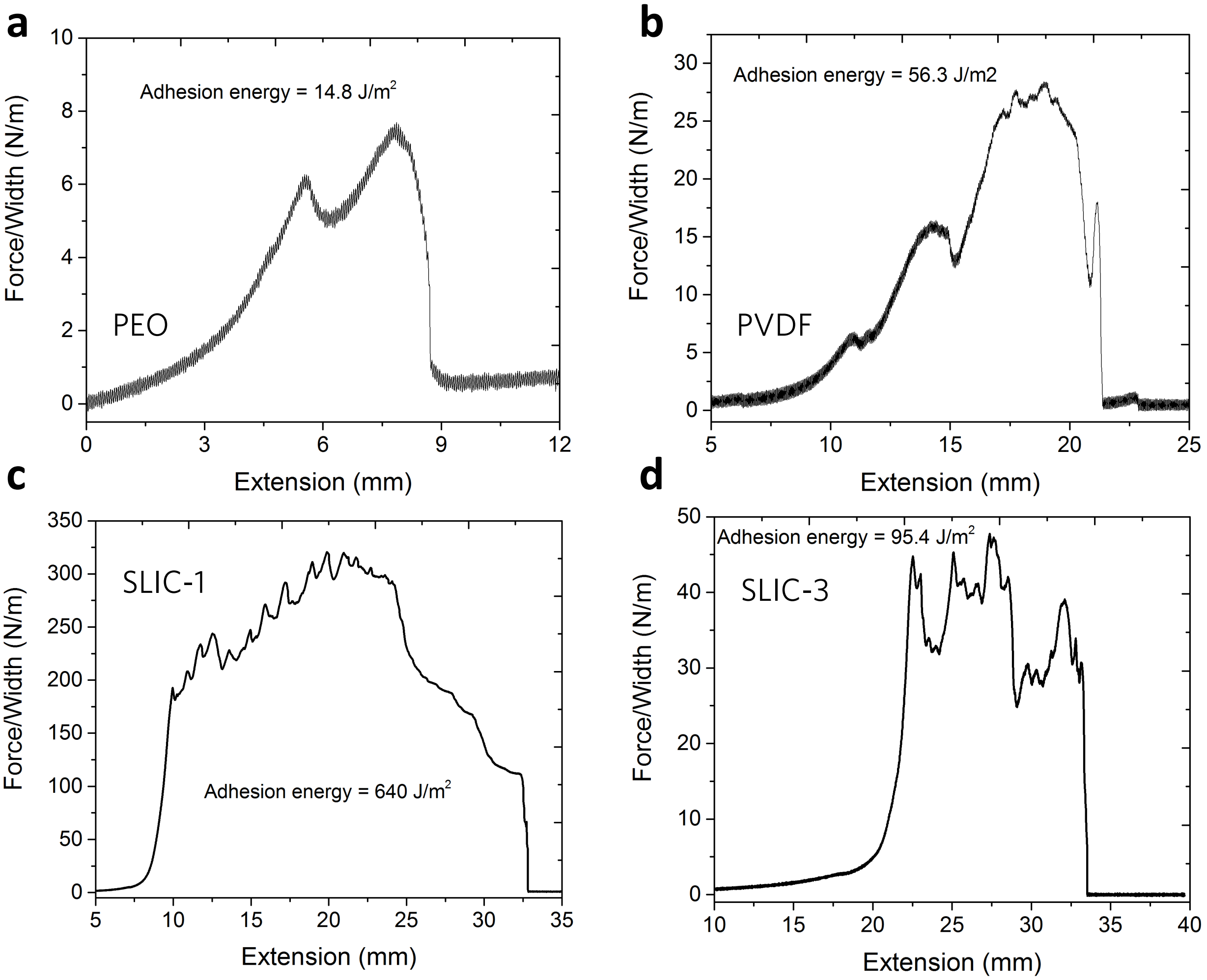

Supplementary Figure 28. Adhesion test of electrode-electrolyte interface. Results of adhesion test between a SLIC-3 electrolyte and electrodes consisting of polymer:LFP:CB with ratio 7:2:1. Different composite electrodes using polymer component of **(a)** PEO, **(b)** PVDF, **(c)** SLIC-1, and **(d)** SLIC-3 were tested.

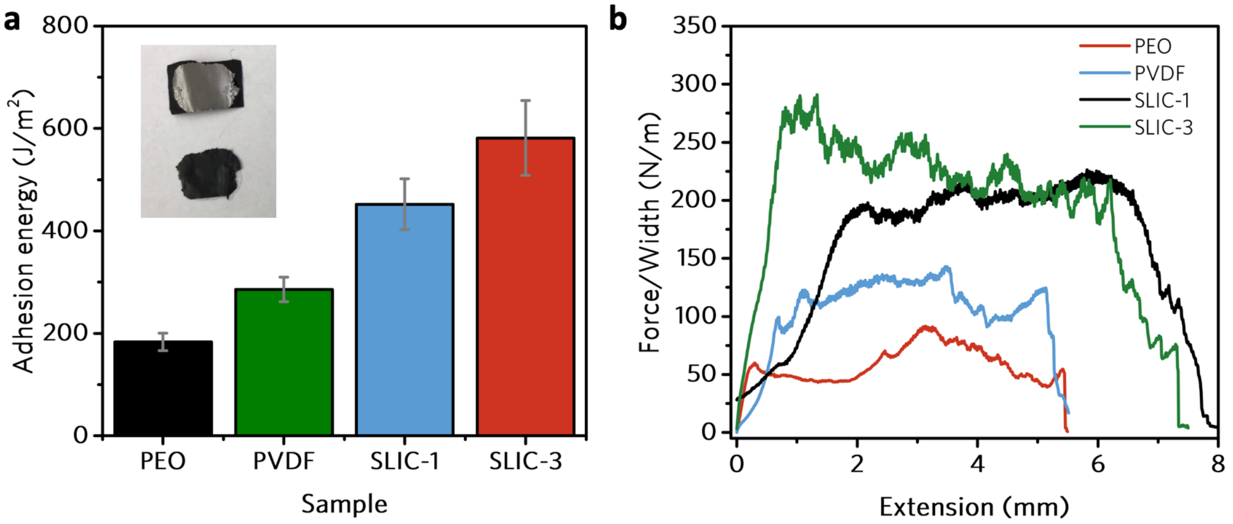

Supplementary Figure 29. Adhesion of different composite electrodes with aluminum current collectors. The composition of all samples is 7:2:1 polymer:LFP:CB. **a** Adhesion energy of the different electrodes. Inset: optical image of one electrode sample after stripping from the aluminum current collector. **b** Normalized force versus extension curves for the peeling of the electrodes from the current collector. It can be seen that the SLIC-based electrodes have higher adhesion energy than either the PVDF or PEO based electrodes. This is due to the increased toughness of the SLIC electrodes compared to the conventional polymers. The increased adhesion energy of the SLIC electrodes makes them promising for applications in stretchable/flexible electronics, where delamination is a serious problem.

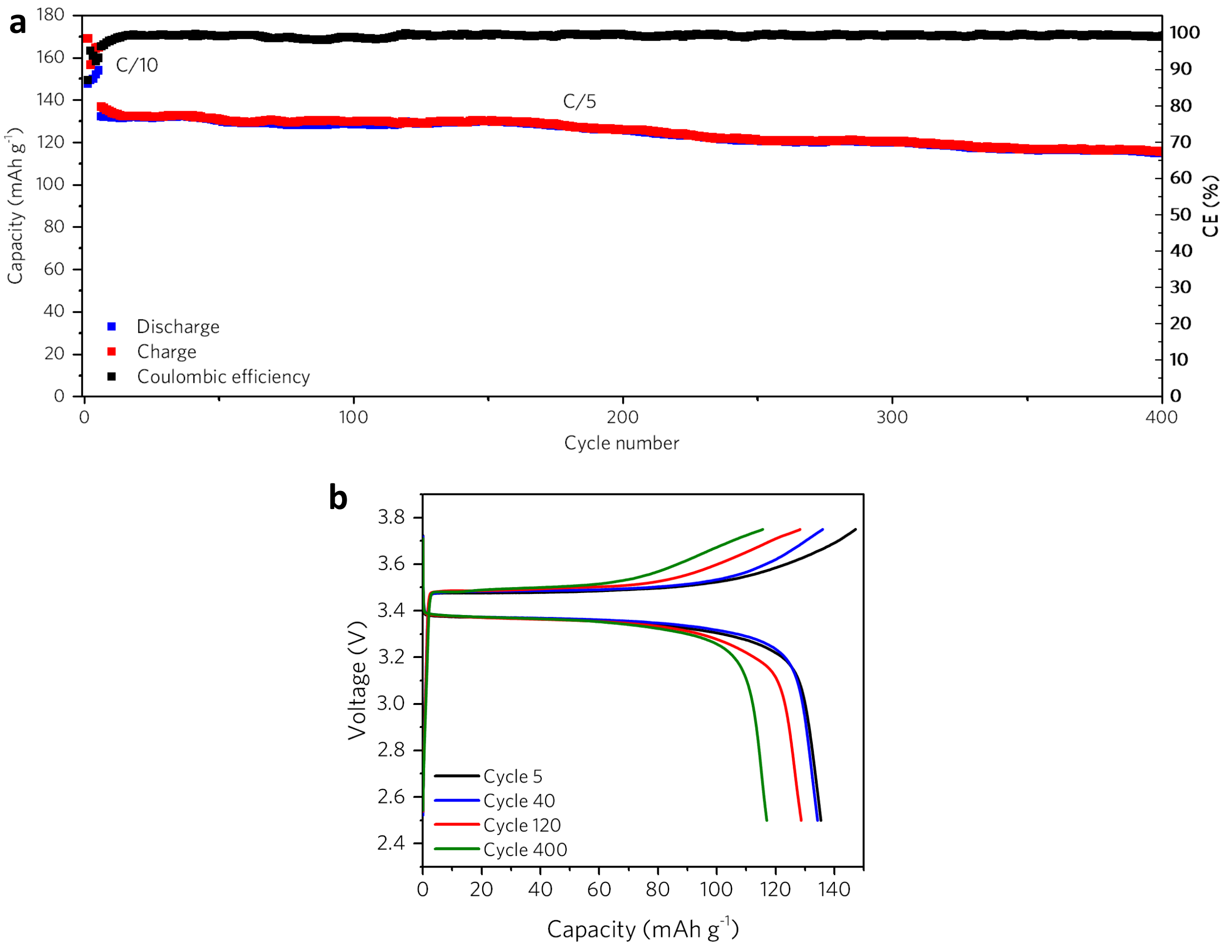

Supplementary Figure 30. a Long-term cycling of a half-cell battery containing a lithium anode, SLIC-3 electrolyte, and SLIC-1:LFP:CB 7:2:1 composite cathode. **b** Charge-discharge curves at different cycle numbers for the battery in. It can be seen that even the electrode material with 70% polymer lasts for over 400 cycles.

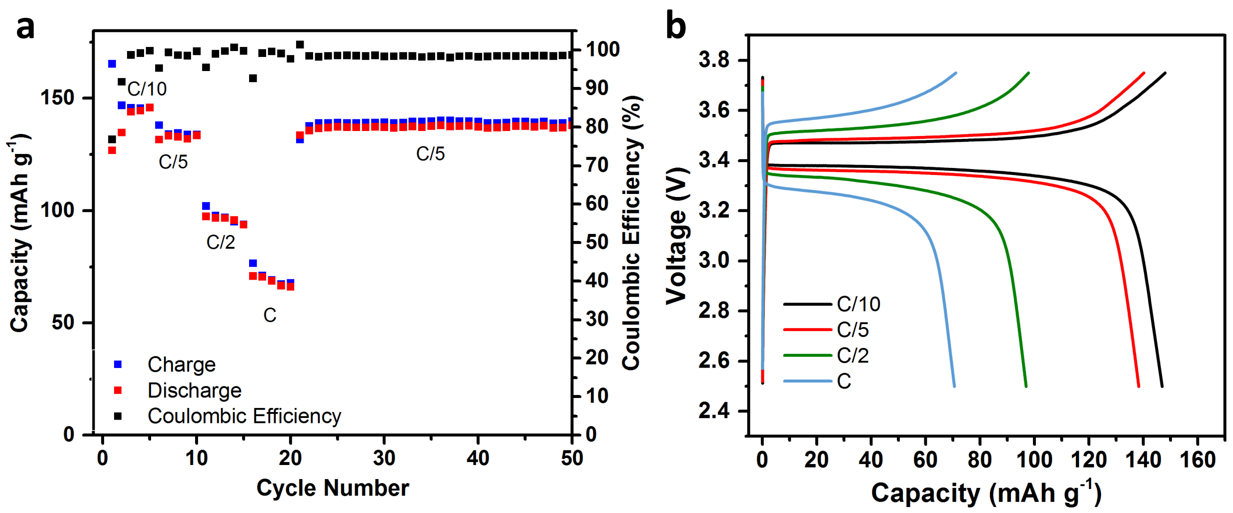

Supplementary Figure 31. Rate capability of half-cells. Electrode composition is 7:2:1 SLIC-1:LFP:CB and electrolyte is the SLIC electrolyte. **a** charge-discharge capacity and coulombic efficiency. **b** Charge-discharge traces for different C-rates. A rate of up to 1C at room temperature can be achieved.

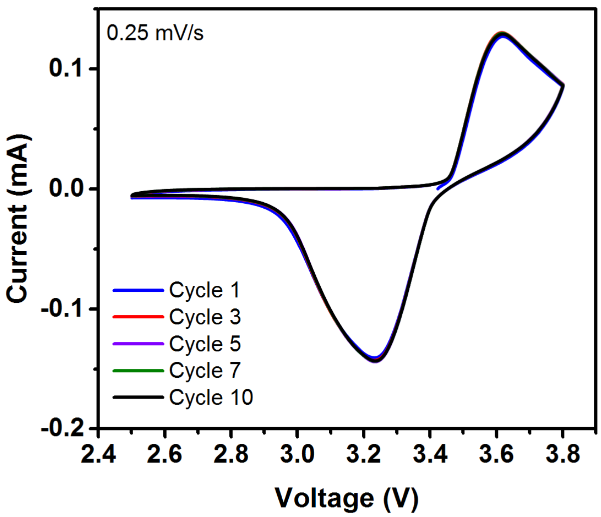

Supplementary Figure 32. Cyclic Voltammogram of LFP||Li Cell. Electrode and electrolyte composition is the same as in Supplementary Figure 25. The scan rate is 0.25 mV/s. No obvious side reactions over the voltage range of interest are observed.

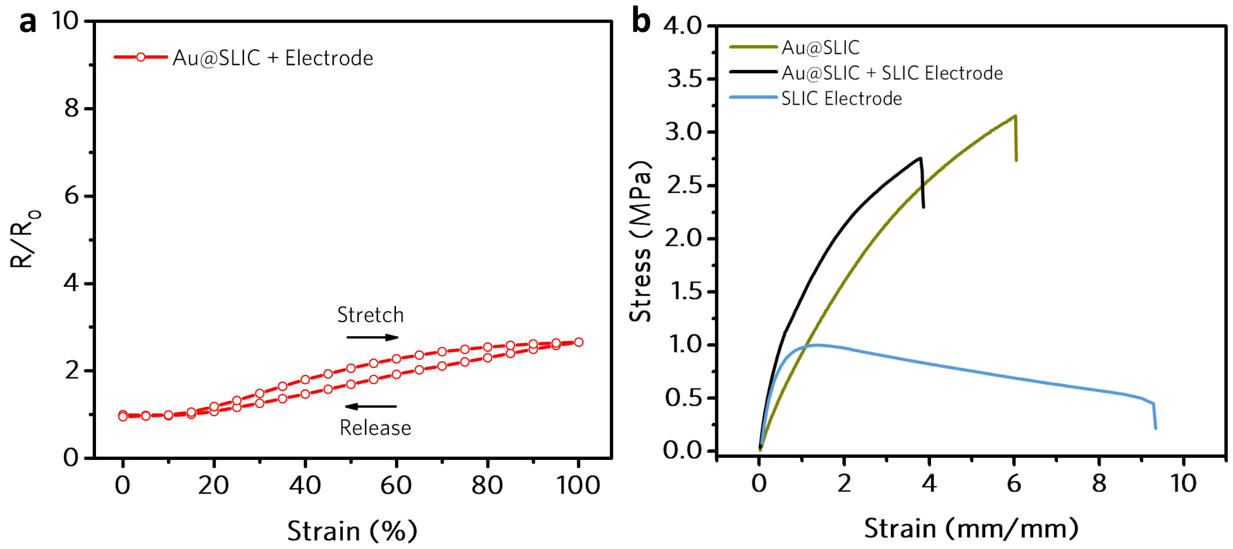

Supplementary Figure 33. Electrical and mechanical performance of the Au@SLIC current collector. **a** resistance as a function of strain. The initial resistance is around 20 Ω □$^{-1}$. **b** Stress-strain curves of the Au@SLIC current collector with and without the SLIC-1 electrode coating. The ratio of the SLIC electrode is 7:2:1 SLIC-1:LFP:CB. The strain rate is 100 mm/min.

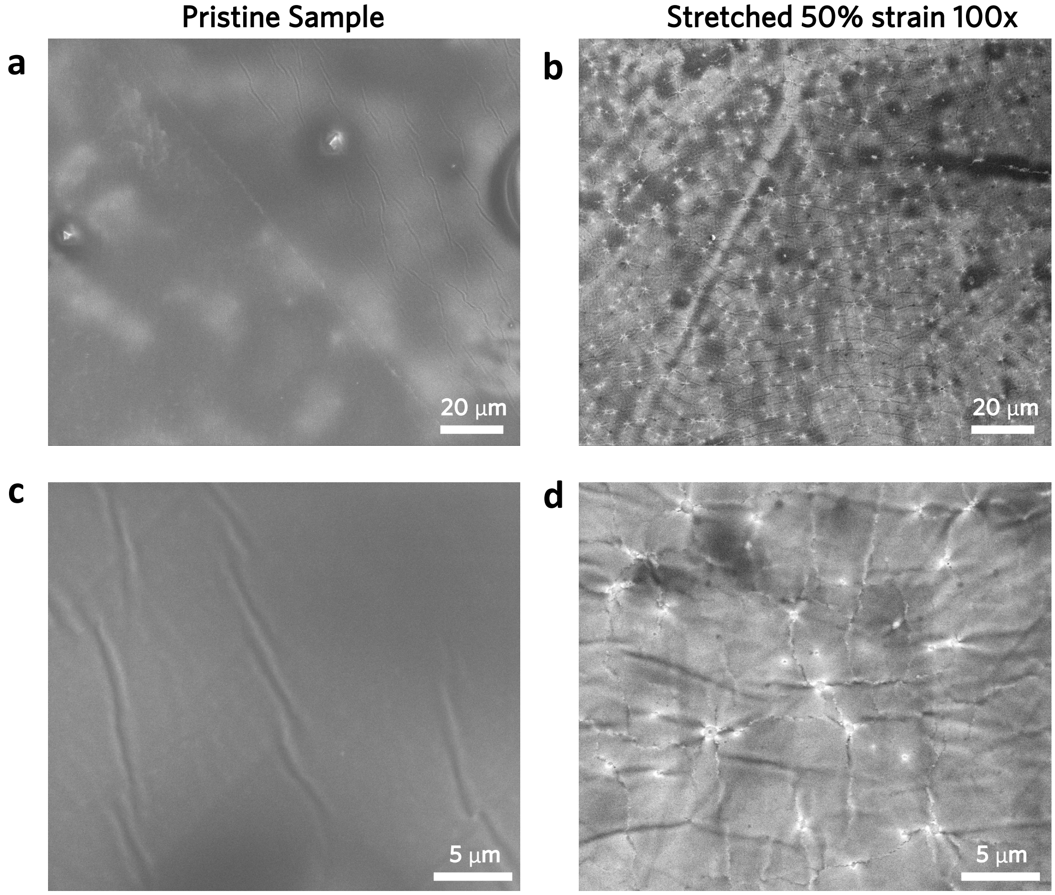

Supplementary Figure 34. Examination of the microcrack formation on the surface of the Au@SLIC electrode upon strain cycling. The Au@SLIC current collector was subjected to 100 cycles at 50% strain. **a** pristine, **b** stretched sample. Images **(c)** and **(d)** show zoomed-in versions of the pristine and stretched samples. It can be seen that the stretching of the current collector causes the formation of microcracked gold. However, these microcracks are interconnected, allowing for a percolation pathway of electrons. Additionally, the SLIC surface appears to form wavy structures upon the stretching process, which contributes to the ability of the current collector to have high conductivity upon stretching. Similar phenomenon have been previously reported for gold-on-elastomer systems.[3]

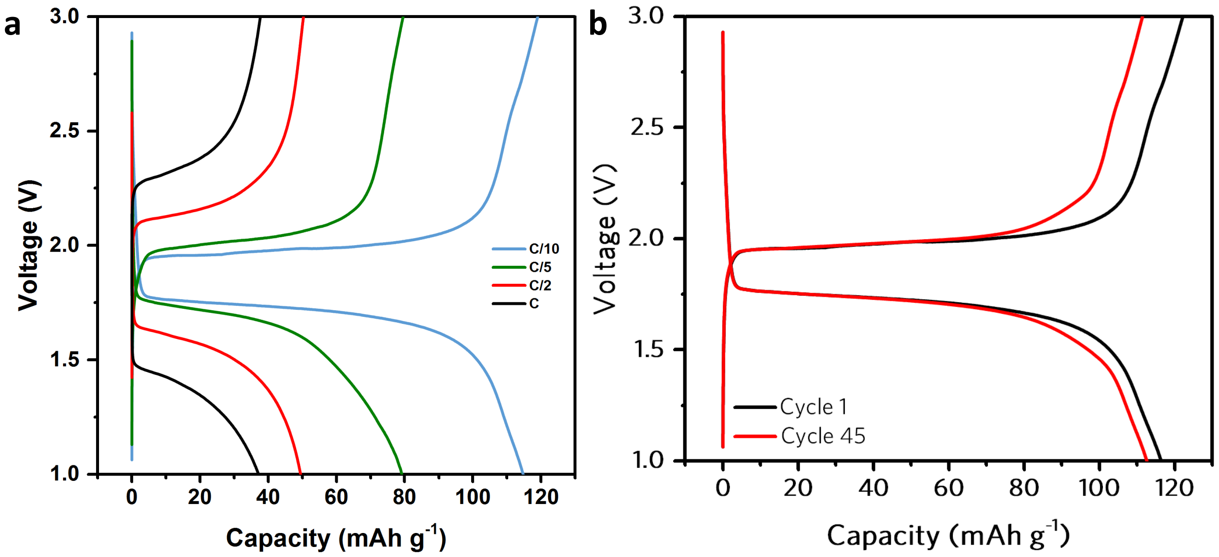

Supplementary Figure 35. Full-cell performance of the stretchable battery with electrode composition of 2:7:1 SLIC-1:LFP/LTO:CB. The SLIC electrolyte is used. Mass loading is 1.1 mAh cm^{-2}. **a** Charge-discharge traces of the full cell at different rates. **b** Charge-Discharge traces of the full cell at cycle 1 (after 2 conditioning cycles) and at cycle 45. Only modest capacity decrease is observed after 45 cycles. The rate is C/10.

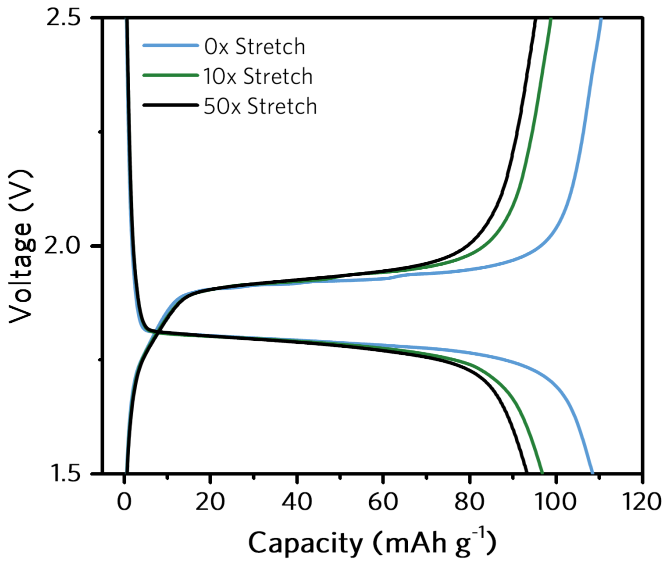

Supplementary Figure 36. Charge-discharge traces of the SLIC battery after 0, 10, and 50 stretching cycles at 50% strain. It can be seen that the largest capacity drop occurs after the first 10 cycles at 50% strain and that there is only marginal capacity fade from the 10th to 50th stretching cycles. This indicates the ability of the SLIC battery to function even when subjected to rigorous mechanical deformation. The composition of the battery is LFP|SLIC-3|LTO. The electrode composition is 2:7:1 SLIC-1:LFP/LTO:CB.

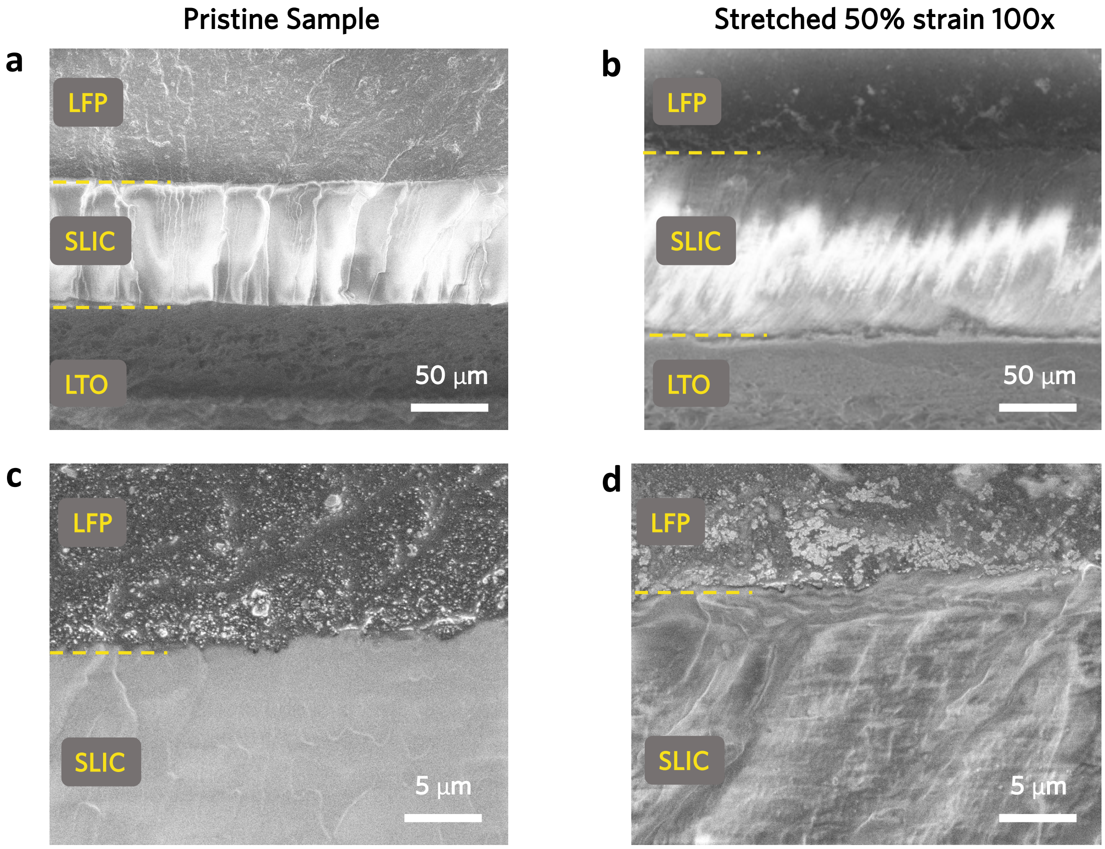

Supplementary Figure 37. Examination of the adhesion between layers of the SLIC battery before and after long-term stretching. The stretching regiment was 100 cycles at 50% strain. The electrode composition is 2:7:1 SLIC-1:Active Material:LFP. **a** Full-stack in the pristine state. **b** Full-stack after stretching. **c** Top (LFP-SLIC) interface before stretching. **d** Top interface after stretching. It can be observed that the electrode-electrolyte interfaces maintain excellent adhesion even after the rigorous stretching cycles. There is some clear deformation in the stretched sample along with minor cracking, however the overall structure of the electrode-electrolyte interfaces remains robust. This result highlights the utility of SLIC to help prevent delamination in stretchable/flexible applications.

Supplementary Tables

Supplementary Table 1. Comparison of the mechanical properties of SLIC materials compared to commercial and previously-reported tough macromolecules.

**Description**	**Young's Modulus (MPa)**	**Ultimate stress (MPa)**	**Extension at Break (mm mm^{-1})**	**Toughness (MJ m^{-3})**	**Ref.**
SLIC-0	0.31 ± 0.11	0.18 ± 0.02	85.8 ± 2.2	3.52 ± 0.54	This work
SLIC-1	0.47 ± 0.08	0.82 ± 0.08	96.2 ± 4.1	52.7 ± 1.2	This work
SLIC-2	0.67 ± 0.12	2.1 ± 0.05	37.3 ± 1.3	55.3 ± 1.1	This work
SLIC-3	5.0 ± 0.43	14.1 ± 0.21	27.1 ± 0.62	205 ± 3.6	This work
SIS Rubber (Sigma Aldrich, 17 wt.% PS)	0.56 ± 0.1	4.4 ± 0.9	32.7 ± 5.2	57.81 ± 2.2	Lab data
PDMS (Sylgard 184)	1.3	5.1	1.58	2.83	[4]
SEBS (Kraton G1643	3	13.5	8.6	48.51	[5]
PDMS-$MPU_{0.4}$-$IU_{0.6}$	0.62	1.7	16.6	13.8	[6]
SPM-3	Not reported	3.8	32.5	70.4	[7]
SPM-2	0.38	0.78	167	117.2	[7]
PT-HM-U10	4.1	39.4	23.5	344.3	[1]
$Zn(Hbimcp)_2$-PDMS	43.7	34.8	24	29.3	[8]
SLIC-3 LiTFSI DEGDME SiO_2	0.58 ± 0.09	2.4 ± 0.12	18.7 ± 1.1	29.3 ± 1.4	This work

Supplementary Table 2. Toughness of Literature-Reported Electrolytes. Assembled partially based on information in a previous publication.[9]

**Description**	**Salt**	**Plasticizer/ Electrolyte**	**σ at RT (S cm^{-1})**	**Young's Modulus (MPa)**	**Ultimate Stress (MPa)**	**Toughness (MJ m^{-3})**	**Ref.**
SLIC-3 + 2wt.% SiO_2	20 wt.% LiTFSI	20% DEGDME	1.2 E-4 ± 2.1 E-5	0.58 ± 0.09	2.4 ± 0.12	29.3 ± 1.4	This Work
SLIC-3 + 2 wt.% SiO_2	20 wt.% LiTFSI	None	5.4 E-7 ± 1.8 E-8	5.2 ± 0.62	11.9 ±0.32	244 ± 3.9	This Work
ePPO	27 wt.% LiTFSI	30 wt.% PC	2.5 E-4	0.3	0.45	0.32	9
PEO	40 wt.% LiTFSI	None	2 E-5	0.4	0.04	0.0463	9
PEO 1 wt. % SiO_2	26 wt.% LiTFSI	None	5.4 E-06	10.7	0.32	0.153	10
Crosslinked PEG Diacrylate	30 wt.% LiTFSI	42 wt.% SCN#	8.0 E-04	1.25	0.062	0.00304	11
Crosslinked PEG Diacrylate	30 wt. % LiTFSI	49 wt.% SCN#	1.0 E-03	4.75	0.095	0.0027	11
Crosslinked PEG Diacrylate High MW	30 wt. % LiTFSI	35 wt.% SCN#	1.1 E-3	0.5	0.24	0.117	12
Aramid fiber in PEO	Not reported	None	5 E-06	4950	61	7.69	13
PEO 20 wt.% LAGP	21 wt.% LiTFSI	None	2.12E-05	14.5	0.58	0.160	10
Celgard 2325	1 M $LiPF_6$	EC:DMC (1:1)	6.2E-04	225	15	1.8	14
ICE	Not reported	None	1.1E-6	Not reported	0.24	1.33	15
PU-PVDF	1 M $LiPF_6$	EC:DEC (1:1)	5.12E-04	Not reported	15.4	10.55	16
PTL-1	10 wt.% LiTFSI	TEGDME	1.1E-4	0.3	1.5	0.585	17
PS-PEO-PS	LiTFSI EO:Li=30	None	1.36E-5*	10	0.077*	0.370*	18

* measured at 60 °C, # SCN = succinonitrile

Stretchable battery comparison

We compare the battery performance of the stretchable SLIC battery demonstrated here. The SLIC based battery is competitive in terms of mass loading, discharge voltage, and stretchability. However, unlike the other reported stretchable batteries, the SLIC battery is intrinsically stretchable. Only one other intrinsically stretchable battery has been reported, but it does not utilize lithium-ion chemistry

Supplementary Table 3. Comparison of the SLIC battery to other stretchable batteries reported in literature. The batteries are compared based on their stretchability, mass loading, and ability to function after strain cycling. It should be noted that while some previously reported stretchable batteries demonstrate excellent performance, the SLIC system is unique in its use of a tough polymer electrolyte as well as being intrinsically stretchable. Furthermore, the SLIC battery demonstrates competitive mass loading and strain capability.

Ref.	Chemistry	Electrolyte	Full-Cell Stretchability	C/C_0* @ max strain	Strain cycles	C/C_0* Post strain cycles	Loading (mAh cm^{-2})	Discharge Voltage	Mechanism of Stretchability
**Non Li-ion**									
19	Zn/Ag_2O	Aqueous Liquid	100%	0.83	10x 100%	0.83	2.5	1.31	Intrinsic - non Li-ion
20	Zn/MnO_2	Viscous Aqueous Gel	100%	0.69	n.r.#	n.r.	3.6	0.85	Microstructured Electrode
21	Zn/MnO_2 Primary Cell	Aqueous Liquid	100%	~1	n.r	n.r.	3.78	1.25	Microstructured Electrode
22	Zn/Ag_2O	Aqueous Liquid	80%	0.82	n.r.	n.r.	0.11	1.5	Nanostructured Electrodes
23	Zn/MnO_2	Aqueous Liquid	50%	0.89	n.r.	n.r.	3.5	1.25	Flowable Electrodes
24	Zn/MnO_2	Flowable Aqueous Gel	75%	~1	700x25%	n.r.	1.44	1.2	Flowable Electrodes
25	Zn/Ag_2O	Aqueous Liquid	100%	~1	500x100%	1	3.5	1.52	Wire shaped
26	Al-Air	Aqueous Gel	30%	n.r.	n.r.	n.r.	n.r.	1.3	Wire shaped
27	Na-Ion	Gel electrolyte	50%	0.88	100x50%	0.89	~0.6	2.6	Microstructured Electrode
**Li-Ion**									
28	$LiMn_2O_4$/LTO	Flowable Organic Gel	100%	0.9	200x100%	0.84	0.0043	2.2	Wire shaped
29	$LiMn_2O_4$/LTO	Flowable Organic Gel	100%	0.85	300x50%	0.99	n.r.	2.5	Wire shaped
30	$LiMn_2O_4$/LTO	Flowable Organic Gel	600%	0.88	100x100%	0.9	n.r.	2.2	Wire shaped
31	LFP/LTO	Organic Liquid	100%	0.85	n.r.	n.r.	0.165	0.85	Microstructured Electrode
32	$LiMn_2O_4$/ Polyimide@AC	Aqueous Liquid	100%	0.8	n.r.	n.r.	0.045	0.95	Microstructured Electrode

16	LCO/Graphite	Organic Liquid	50%	0.91	60x50%	0.85	2.2	3.6	Wavy Structure
33	$LiMn_2O_4$/LTO	Flowable Organic Gel	400%	0.99	200x400%	0.97	0.12	1.55	Wavy Structure
34	LCO/LTO	Flowable Organic Gel	300%	n.r.	n.r.	n.r.	1.15	2.3	Rigid Islands
35	Li-Air	Gel electrolyte	100%	n.r.	1000x75%	n.r.	n.r.	2	Rigid Islands + Wavy Structure
36	LCO/Graphite	Organic Liquid	150%	~1	100x83%	n.r.	5.8	3.6	Kirigami Folding
37	LCO/LTO	Organic Liquid	1300%	~1	50x1300%	~1	0.2	2.6	Origami Folding
**This Work**	**LFP/LTO**	**Polymer Electrolyte**	**70%**	**0.92**	**50x50%**	**0.86**	**1.1**	**1.8**	**Intrinsic**

*C/C_0 refers to the capacity retention of the strained battery compared to the pristine one
#n.r. = not reported

Supplementary Table 4. Properties of commercially available flexible batteries obtained via internet search.

Provider	Chemistry	Electrolyte	Loading (mAh cm-2)	Discharge Voltage	Primary/Secondary	Link
Blue Spark	Zn / MnO_2	unknown	1.1	1.5	Primary	Blue Spark
Power Stream	Li-Polymer	polymer	~2	3.6	Secondary	PowerStream
BrightVolt	Li Metal	polymer	2.4	< 3	secondary (in development)	BrightVolt
Jenax	Li-Ion	polymer gel	2.3	3.8	secondary (in development)	Jenax

Supplementary Notes

Toughness (U_T) is defined as the area under the stress (σ) strain (ε) curve until the fracture strain (ε_f) is reached (Equation 1). [38]

$$U_T = \int_0^{\varepsilon_f} \sigma d\varepsilon \quad (1)$$

Supplementary Methods

Synthesis of SLIC materials

All reagents were commercially available and used as supplied without further purification. Deuterated solvents were purchased from Acros Organics. Poly(propylene glycol)-*block*-poly(ethylene glycol)-*block*-poly(propylene glycol) (PPG-PEG-PPG, M_n = 2000), isophorone diisocyanate (IPDI), 1,5-pentanediol (PD) were purchased from Sigma-Aldrich (USA) and dried under vacuum at 80 °C overnight before use. Dibutyltin dilaurate (DBTDL) catalyst was purchased from Alfa Aesar. Prepolymer **1**[39] and 5-(2-hydroxyethyl)-6-methyl-2-aminouracil **2**[40] were prepared according to the published procedures. ^{1}H NMR spectra were recorded on a Varian Mercury 400 NMR spectrometer at room temperature with use of the deuterated solvent as the lock and the residual solvent or TMS as the internal reference. ^{7}Li NMR spectra were collected using an Inova 300 MHz spectrometer. Polymer/salt/additive mixtures were dissolved to a concentration of 5 wt.% in deuterated chloroform ($CDCl_3$), and placed into 5 mm borosilicate NMR tubes. $CDCl_3$ does interfere with solvation of LiTFSI as indicated by the lack of ^{7}Li peak in a neat LiTFSI-$CDCl_3$ mixture. In all instances, samples were prepared and tightly sealed in NMR tubes in the nitrogen environment. Gel permeation chromatography (GPC) was carried out in DMF on two PolyPore columns (Agilent) connected in series with a DAWN multiangle laser light scattering (MALLS) detector and an Optilab TrEX differential refractometer (both from Wyatt Technology).

<u>Synthesis of prepolymer **1**</u>: In a 250 mL three-necked flask equipped with mechanical stirrer were added PPG-PEG-PPG (10.0 g, 5.00 mmol) and IPDI (3.33 g, 15 mmol) under nitrogen atmosphere. Three drops of DBTDL catalyst was then added and the resultant mixture was kept stirring at 85

°C for 3 h to yield prepolymer **1**. Afterwards, 10 mL of dry DMF was added to dissolve prepolymer **1**.

Synthesis of SLIC-**0**: Chain extender PD (1.04 g, 10.0 mmol) in 10 mL of dry DMF was added to the prepolymer solution. With fully stirring at 85 °C for another 3 h, methanol (2 mL) was added and the mixture was further stirred for 30 min to ensure all isocyanate groups were consumed. Then, the mixture was poured into a PTFE plate which was subsequently put in a vacuum oven at 90 °C for 12 h to allow the reaction to complete. By dissolving the crude polymer in 100 mL of THF and then precipitating it into the mixture of acetonitrile/diethyl ether (1:4, *v*/*v*), we successfully obtained purified SLIC-**0** (9.34 g, 65%) as a pale-yellow elastomer. The purification process was repeated three times. GPC using DMF as the eluent measured the average molecular weight as M_n = 100 KDa. The ^{1}H NMR spectrum of SLIC-**0** is shown in Figure S1. ^{1}H NMR ($CDCl_3$, room temperature, 400 MHz) δ (ppm): 4.87 (s), 4.60 (s), 3.94–4.12 (m), 3.23–3.87 (m), 2.89 (d), 1.53–1.76 (m), 1.32–1.45 (m), 0.76–1.28 (m).

Syntheses of SLIC-**1**: Chain extenders PD (0.88 g, 8.00 mmol) and compound **2** (0.34 g, 2.00 mmol) in 10 mL of dry DMSO were added to the prepolymer solution. With fully stirring at 85 °C for another 3 h, methanol (2 mL) was added and the mixture was further stirred for 30 min to ensure all isocyanate groups were consumed. Then, the mixture was poured into a PTFE plate which was subsequently put in a vacuum oven at 90 °C for 12 h to allow the reaction to complete. By dissolving the crude polymer in 100 mL of THF and then precipitating it into acetonitrile/diethyl ether (1:4, *v*/*v*), we successfully obtained purified SPM-**1** (10.0 g, 69%) as a pale-yellow elastomer. The purification process was repeated three times. GPC using DMF as the eluent measured the average molecular weight as M_n = 104 KDa. The ^{1}H NMR spectrum of SLIC-**1** is shown in Figure S2. ^{1}H NMR ($CDCl_3$, room temperature, 400 MHz) δ (ppm): 12.90 (s), 11.95 (s), 10.11 (s), 4.87 (s), 4.56 (s), 3.96–4.13 (m), 3.15–3.87 (m), 2.89 (d), 2.25 (s), 1.51–1.79 (m), 1.33–1.47 (m), 0.62–1.32 (m). The ratio of the methyl signal on UPy (highlighted in purple) and ethyl protons of the PD unit (highlighted in green) confirms the incorporation of the corresponding 20 mol% of noncovalent UPy cosslinkers into the polymeric backbone. The same calculation method is also used for SLICs **2** and **3** to confirm that the amounts of UPy crosslinkers incorporated in the polymeric backbones are consistent with the theoretical values.

Syntheses of SLIC-**2**: Chain extenders PD (0.42 g, 4.00 mmol) and compound **2** (1,02 g, 6.00 mmol) in 15 mL of dry DMSO were added to the prepolymer solution. With fully stirring at 85 °C for another 3 h, methanol (2 mL) was added and the mixture was further stirred for 30 min to ensure all isocyanate groups were consumed. Then, the mixture was poured into a PTFE plate which was subsequently put in a vacuum oven at 90 °C for 12 h to allow the reaction to complete. By dissolving the crude polymer in 150 mL of THF and then precipitating it into acetonitrile/diethyl ether (1:4, *v*/*v*), we successfully obtained purified SLIC-**2** (9.60 g, 65%) as a pale-yellow elastomer. The purification process was repeated three times. GPC using DMF as the eluent measured the average molecular weight as M_n = 85.5 KDa. The ^{1}H NMR spectrum of SLIC-**2** is shown in Figure S3. ^{1}H NMR ($CDCl_3$, room temperature, 400 MHz) δ (ppm): 12.92 (s), 11.98 (s), 10.12 (s), 4.87 (s), 4.58 (s), 3.24–3.83 (m), 2.89 (d), 2.27 (s), 1.54–1.77 (m), 1.33–1.45 (m), 0.73–1.30 (m).

Syntheses of SLIC-**3**: Chain extender **2** (1.69 g, 10.0 mmol) in 20 mL of dry DMSO were added to the prepolymer solution. With fully stirring at 85 °C for another 3 h, methanol (2 mL) was added and the mixture was further stirred for 30 min to ensure all isocyanate groups were consumed. Then, the mixture was poured into a PTFE plate which was subsequently put in a vacuum oven at 90 °C for 12 h to allow the reaction to complete. By dissolving the crude polymer in 200 mL of THF and then precipitating it into acetonitrile/diethyl ether (1:4, *v*/*v*), we successfully obtained purified SLIC-**3** (10.7 g, 71%) as a pale-yellow elastomer. The purification process was repeated three times. GPC using DMF as the eluent measured the average molecular weight as M_n = 92.0 KDa. The ^{1}H NMR spectrum of SLIC-**3** is shown in Figure S4. ^{1}H NMR ($CDCl_3$, room temperature, 400 MHz) δ (ppm): 12.89 (s), 11.95 (s), 10.06 (s), 4.87 (s), 4.55 (s), 3.06–3.85 (m), 2.89 (d), 2.27 (s), 1.50–1.78 (m), 0.47–1.37 (m).

Physical characterization of SLIC materials

DSC experiments were carried out with a TA Instruments DSC Q2000 using Tzero Aluminum pans. The DSC procedure involved two heating and cooling cycles from -90 °C to 140 °C at a scan rate of 10 K min^{-1}. Data was analyzed using the T_g finder in the TA Universal Analysis software

package. Glass transition data were determined based on the value obtained from the second heating trace.

Mechanical tensile-stress and adhesion strength experiments were performed using an Instron 5848 Microtester with a 10N force transducer. Typically, rectangular samples with dimensions of 5 mm by 2 mm by 0.2 mm were cut and fixed between two hand-tightened clamps. Strain rates were 100 mm/min for extensibility tests and 30 mm min^{-1} for strain cycling tests.

Adhesion measurements were carried out using a Instron 5848 Microtester with a 10N force transducer. One side of each sample was adhered to a thin (50 μm) piece of Kapton film with two-part epoxy. The sample size was roughly 1 cm by 1 cm. The two sides to be adhered were then affixed to each other with 1 hour of mechanical pressure from a binder clip. For the experiments measuring the interfacial adhesion between the current collector and the electrode, the electrode was first cast onto the current collector, and then the Kapton was adhered to either side. Adhesion energies were calculated using a previously reported method.[41]

Creep measurements were performed using a TA DMA Q800. In these experiments, a 7 mm by 2 mm by 0.2 mm rectangular sample was cut and fixed between hand-tightened clamps. A stress of either 0.5 MPa or 0.1 MPa was applied for 15 minutes. Upon release of the stress, the relaxation of the sample was observed for an additional 30 minutes.

Rheological experiments were carried out using a stress-controlled rheometer (TA Instruments Model AR-G2) with a 8 mm parallel plate attachment. All the samples were performed dynamic amplitude sweep experiments to confirm their linear region. Master curves were created using time-temperature superposition (TTS). The frequency sweep was performed at temperatures between 100 °C and 20 °C with an interval of 20 °C over the frequency range between 0.628 and 628 rad/s. The reference temperature is 20 °C, and a strain of 1% was used.

The small-angle x-ray scattering (SAXS) measurements on polymer films in transmission geometry were carried out on beamline 4-2 at Stanford Synchrotron Radiation Lightsource (SSRL) of SLAC National Accelerator Laboratory (SLAC). The standalone film (~0.5mm) was illuminated using a 15 keV X-ray beam. A Rayonix MX225-HE detector at a sample-to-detector distance of 3.0 m was equipped to record the scattering intensity. Data analysis was performed using the Nika and Irena software packages for Igor Pro software.[42]

Field emission scanning electron microscope (FESEM) (JEOL JSM-7600F) was employed to observe the surface morphology under 5 kV gun voltage. SEM images for cross-section samples

utilized a cross-sectional holder and samples were cut with a razor blade under cryogenic conditions to minimize influence of cutting on the sample morphology.

FTIR spectra were measured using a Nicolet iS50 FT/IR Spectrometer (Thermo Fisher) with a diamond attenuated total reflectance (ATR) attachment. A small amount of sample (~10 mg) was placed onto the stage and firmly clamped in place. A total of 32 measurements with a resolution of 2 cm^{-1} were collected for each sample. Spectra were processed and baseline corrected using the OMNIC software package.

Fabrication of SLIC electrolytes

SLIC polymers (0.54 g) were dissolved in 11 mL of THF along with an appropriate amount of vacuum-dried LiTFSI and 14nm fumed SiO_2. The SiO_2 nanoparticles were obtained from Sigma Aldrich (USA) and were prepared via flame pyrolysis of $SiCl_4$ in hydrogen and oxygen. The particles are hydroxy-terminated on the surface, allowing hydrogen bonding to occur. After dispersing, the viscous solution was degassed and cast into a Teflon mold, and dried for 24 hours at RT. After drying at RT, the film was further dried for 24 hours at 60 °C in a vacuum oven and for 24 hours in a nitrogen filled glovebox. Resulting films were 20-200 µm thick. To use, the films were peeled, punched, and plasticized in the confines of the nitrogen glovebox. The electrolytes were plasticized by addition of a defined amount of DEGMDE with a micropipette. The electrolytes were then allowed to swell with the added plasticizer for 1 hour prior to use.

Fabrication of SLIC electrodes

SLIC polymer (250mg) and LiTFSI (63 mg) were dissolved in N-methyl-2-pyrrolidone (500 uL) to make a viscous liquid. Active material (LFP/LTO, MTI), and carbon black (Timcal SuperP) were then added in appropriate weight ratios ranging from 7:2:1 polymer:LFPLCB to 2:7:1. The amount of carbon black remained fixed at 10 wt.%. The slurries were mixed using a dual asymmetric centrifugal mixer (FlackTek). Resulting slurries were doctor bladed onto either a Teflon block or current collector and then dried for 12 hours at RT and 24 hours at 70 °C under vacuum. Films were rapidly transferred into a nitrogen-filled glovebox, peeled, and then cut to the appropriate size.

Electrochemical characterization

All electrochemical measurements were preformed using a Biologic VSP-300 potentiostat. Temperature controlled experiments utilized a Espec environmental chamber. Electrochemical impedance measurements were conducted by sandwiching polymer films in a symmetric stainless steel (SS||SS) coin cell. A Teflon spacer of 150 μm was used to ensure no thickness change during the measurement. A frequency range of 7 MHz to 100 mHz with a polarization amplitude of 50 mV was used. Temperature-dependent ionic conductivity was measured from 0 to 70 °C with equilibration time of 1 hour at each temperature. Strain-dependent ionic conductivity was conducted by connecting the potentiostat into the glovebox and measuring the impedance between two stainless steel disks clamped onto the stretched polymer film with a fixed amount of pressure. For other electrochemical tests, samples were transferred hermetically to an argon filled glovebox. Electrochemical stability was probed using a Li||SS cell at 40 °C over a range of 0 to 4 V with a scan rate of 0.25 mV/s . Lithium transference number was calculated using a Li||Li symmetric cell at 40 °C with a polarization of 50 mV (see SI for details).

Non-stretchable battery tests were conducted using an Arbin battery cycler in 2032 coin cells. A 2cm^2 disk of plasticized electrolyte was placed on top of a freshly scraped 1 cm^2 Li disk. A 1 cm^2 composite electrode coated onto an Al current collector was placed on top of the electrolyte and the stack was sealed in the coin cell. Battery cycling was conducted using galvanically-controlled, potentionstatic-limited cycling. For the LFP||Li half cells, an upper cutoff voltage of 3.8 V vs. Li/Li$^+$ was used and a lower voltage cutoff of 2.5 V vs Li/Li$^+$ was used. For LFP||LTO full cells, an upper cutoff of 3 V vs. Li/Li$^+$ was used and a lower cutoff of 1 V vs. Li/Li$^+$ was used. The rates of C/10, C/5, C/2, and C were calculated based on the mass of active material present in the sample and the theoretical capacity of the active material.

Fabrication of stretchable batteries

Stretchable current collectors were fabricated by thermal evaporation of a gold film of 40-100 nm onto a thin (20 μm) film of SLIC-3. The evaporation rate was 8 Å s^{-1}. The strain-dependence of electronic resistance was measured using a customized stretcher and resistance monitor (Agilent E4980A precision LCR Meter). To make stretchable batteries, the composite electrode slurry was doctor-bladed with a gap height of 10-200 μm directly onto the Au@SLIC film. Following drying in the vacuum glovebox at 60 °C, Au@SLIC+electrode slurries were transferred into the nitrogen filled glovebox. In the glovebox, the SLIC electrolyte was plasticized, and the components were

assembled in the following order: Au@SLIC+LTO || SLIC electrolyte || Au@SLIC+LFP. Aluminum tabs were taped to the edge of the Au@SLIC current collectors, and the entire stack was sandwiched between two slabs of PDMS (EcoFlex DragonSkin 10 Medium) and sealed with a coating of liquid PDMS. Following overnight curing, the battery was transferred out of the glovebox and probed electrochemically. For long-term cycling measurements, stretchable battery components were sealed in coin-cells to reduce the moisture permeability. Typical stretchable batteries had an active material area of 1 cm^2. For the LED demonstration, two stretchable batteries with an active material area of 1 cm^2 were connected in parallel after sealing in PDMS.

Calculation of error

Error bars, where present, are calculated based on the standard deviation of multiple measurements. All experiments were conducted in multiple measurements, most commonly n>3. Note that for battery measurements, standard deviations are not commonly reported. We have selected a 'representative' performance from multiple cells for each experiment.

Supplementary References

1. Song, Y., Liu, Y., Qi, T. & Li, G. L. Towards Dynamic but Supertough Healable Polymers through Biomimetic Hierarchical Hydrogen-Bonding Interactions. *Angew. Chem. Int. Ed.* **57**, 13838–13842 (2018).
2. Evans, J., Vincent, C. A. & Bruce, P. G. Electrochemical measurement of transference numbers in polymer electrolytes. *Polymer* **28**, 2324–2328 (1987).
3. Matsuhisa, N., Chen, X., Bao, Z. & Someya, T. Materials and structural designs of stretchable conductors. *Chem. Soc. Rev.* **48**, 2946–2966 (2019).
4. Johnston, I. D., McCluskey, D. K., Tan, C. K. L. & Tracey, M. C. Mechanical characterization of bulk Sylgard 184 for microfluidics and microengineering. *J. Micromech. Microeng.* **24**, (2014).
5. Cooper, C. B. *et al.* Toughening stretchable fibers via serial fracturing of a metallic core. *Sci. Adv.* **5**, 1–9 (2019).
6. Kang, J. *et al.* Tough and Water-Insensitive Self-Healing Elastomer for Robust Electronic Skin. *Adv. Mater.* **30**, 1–8 (2018).
7. Yan, X. *et al.* Quadruple H-Bonding cross-linked supramolecular polymeric materials as substrates for stretchable, antitearing, and self-healable thin film electrodes. *J. Am. Chem. Soc.* **140**, 5280–5289 (2018).
8. Lai, J. C. *et al.* Thermodynamically stable whilst kinetically labile coordination bonds lead to strong and tough self-healing polymers. *Nat. Commun.* **10**, 1–9 (2019).
9. Lopez, J. *et al.* A Dual-Crosslinking Design for Resilient Lithium-Ion Conductors. *Adv. Mater.* **1804142**, 1–9 (2018).
10. Chen, S., Zhao, Y., Yang, J., Yao, L. & Xu, X. Hybrid solid electrolytes with excellent

electrochemical properties and their applications in all-solid-state cells. *Ionics* **23**, 2603–2611 (2017).
11. Echeverri, M., Hamad, C. & Kyu, T. Highly conductive, completely amorphous polymer electrolyte membranes fabricated through photo-polymerization of poly(ethylene glycol diacrylate) in mixtures of solid plasticizer and lithium salt. *Solid State Ionics* **254**, 92–100 (2014).
12. He, R., Echeverri, M., Ward, D., Zhu, Y. & Kyu, T. Highly conductive solvent-free polymer electrolyte membrane for lithium-ion batteries: Effect of prepolymer molecular weight. *J. Memb. Sci.* **498**, 208–217 (2016).
13. Tung, S.-O., Ho, S., Yang, M., Zhang, R. & Kotov, N. A. A dendrite-suppressing composite ion conductor from aramid nanofibres. *Nat. Commun.* **6**, 6152 (2015).
14. Cannarella, J. *et al.* Mechanical Properties of a Battery Separator under Compression and Tension. *J. Electrochem. Soc.* **161**, F3117–F3122 (2014).
15. Shi, L. *et al.* Highly stretchable and transparent ionic conducting elastomers. *Nat. Commun.* **9**, 2630 (2018).
16. Liu, W. *et al.* Stretchable Lithium-Ion Batteries Enabled by Device-Scaled Wavy Structure and Elastic-Sticky Separator. *Adv. Energy Mater.* **7**, 1–6 (2017).
17. Porcarelli, L., Gerbaldi, C., Bella, F. & Nair, J. R. Super Soft All-Ethylene Oxide Polymer Electrolyte for Safe All-Solid Lithium Batteries. *Sci. Rep.* **6**, 19892 (2016).
18. Bouchet, R. *et al.* Single-ion BAB triblock copolymers as highly efficient electrolytes for lithium-metal batteries. *Nat. Mater.* **12**, 452–7 (2013).
19. Kumar, R. *et al.* All-Printed, Stretchable Zn-Ag2O Rechargeable Battery via Hyperelastic Binder for Self-Powering Wearable Electronics. *Adv. Energy Mater.* **7**, (2017).
20. Zhu, H. W. *et al.* Dip-coating processed sponge-based electrodes for stretchable Zn-MnO2batteries. *Nano Res.* **11**, 1554–1562 (2018).
21. Gaikwad, A. M. *et al.* Highly stretchable alkaline batteries based on an embedded conductive fabric. *Adv. Mater.* **24**, 5071–5076 (2012).
22. Yan, C. *et al.* Stretchable silver-zinc batteries based on embedded nanowire elastic conductors. *Adv. Energy Mater.* **4**, 1–6 (2014).
23. Kaltenbrunner, M., Kettlgruber, G., Siket, C., Schwödiauer, R. & Bauer, S. Arrays of ultracompliant electrochemical dry gel cells for stretchable electronics. *Adv. Mater.* **22**, 2065–2067 (2010).
24. Kettlgruber, G. *et al.* Intrinsically stretchable and rechargeable batteries for self-powered stretchable electronics. *J. Mater. Chem. A* **1**, 5505–5508 (2013).
25. Zamarayeva, A. M. *et al.* Flexible and stretchable power sources for wearable electronics. *Sci Adv* **3**, e1602051 (2017).
26. Xu, Y., Zhao, Y., Ren, J., Zhang, Y. & Peng, H. An All-Solid-State Fiber-Shaped Aluminum–Air Battery with Flexibility, Stretchability, and High Electrochemical Performance. *Angew. Chem. Int. Ed.* **55**, 7979–7982 (2016).
27. Li, H. *et al.* An All-Stretchable-Component Sodium-Ion Full Battery. *Adv. Mater.* **29**, 1700898 (2017).
28. Ren, J. *et al.* Elastic and wearable wire-shaped lithium-ion battery with high electrochemical performance. *Angew. Chem. Int. Ed.* **53**, 7864–7869 (2014).
29. Zhang, Y. *et al.* Flexible and stretchable lithium-ion batteries and supercapacitors based on electrically conducting carbon nanotube fiber springs. *Angew. Chem. Int. Ed.* **53**, 14564–14568 (2014).

30. Zhang, Y. *et al.* Super-stretchy lithium-ion battery based on carbon nanotube fiber. *J. Mater. Chem. A* **2**, 11054–11059 (2014).
31. Shin, M. *et al.* Highly Stretchable Separator Membrane for Deformable Energy-Storage Devices. *Adv. Energy Mater.* **1801025**, 1–10 (2018).
32. Song, W. J. *et al.* Jabuticaba-Inspired Hybrid Carbon Filler/Polymer Electrode for Use in Highly Stretchable Aqueous Li-Ion Batteries. *Adv. Energy Mater.* **8**, 1–10 (2018).
33. Weng, W. *et al.* A gum-like lithium-ion battery based on a novel arched structure. *Adv. Mater.* **27**, 1363–1369 (2015).
34. Xu, S. *et al.* Stretchable batteries with self-similar serpentine interconnects and integrated wireless recharging systems. *Nat. Commun.* **4**, 1543–1548 (2013).
35. Wang, L., Zhang, Y., Pan, J. & Peng, H. Stretchable lithium-air batteries for wearable electronics. *J. Mater. Chem. A* **4**, 13419–13424 (2016).
36. Song, Z. *et al.* Kirigami-based stretchable lithium-ion batteries. *Sci. Rep.* **5**, 1–9 (2015).
37. Song, Z. *et al.* Origami lithium-ion batteries. *Nat. Commun.* **5**, 1–6 (2014).
38. Callister Jr., W. D. & Rethwisch, D. G. *Fundamentals of materials science and engineering: an integrated approach*. (John Wiley & Sons, Inc., 2012).
39. Zhang, Q. *et al.* Synthesis of N-phenylaminomethyl POSS and its utilization in polyurethane. *Macromolecules* **44**, 550–557 (2011).
40. Gangjee, A. *et al.* Design, synthesis, and x-ray crystal structure of a potent dual inhibitor of thymidylate synthase and dihydrofolate reductase as an antitumor agent. *J. Med. Chem.* **43**, 3837–3851 (2000).
41. Li, J. *et al.* Tough adhesives for diverse wet surfaces. *Science* **357**, 378–381 (2017).
42. Ilavsky, J. Nika : software for two-dimensional data reduction. *J. Appl. Crystallogr.* **45**, 324–328 (2012).
