# Supplementary material for: Decoupling of mechanical properties and ionic conductivity in supramolecular lithium ion conductors
Source: Nat Commun. 2019 Nov 26;10:5384. doi: 10.1038/s41467-019-13362-4 (PMC6879760; doi:10.1038/s41467-019-13362-4)
Supplement: Supplementary file 2 — Description of Additional Supplementary Files [file 41467_2019_13362_MOESM2_ESM.pdf]

Description of Additional Supplementary Files

Supplementary Movie 1: Stretchable battery undergoing 70% strain and folding and bending. While providing constant power output to an LED.
